# *DRD4* 48 bp multiallelic variants as age-population-specific biomarkers in attention-deficit/hyperactivity disorder

**DOI:** 10.1038/s41398-020-0755-4

**Published:** 2020-02-19

**Authors:** Cristian Bonvicini, Samuele Cortese, Carlo Maj, Bernhard T. Baune, Stephen V. Faraone, Catia Scassellati

**Affiliations:** 1 Molecular Markers Laboratory, IRCCS Istituto Centro San Giovanni di Dio Fatebenefratelli, Brescia, Italy; 2 Center for Innovation in Mental Health, Academic Unit of Psychology, University of Southampton, Southampton, SO17 1BJ UK; 3 Clinical and Experimental Sciences (CNS and Psychiatry), Faculty of Medicine, University of Southampton, Southampton, SO17 1BJ UK; 4 Solent NHS Trust, Southampton, SO19 8BR UK; 5 New York University Child Study Center, New York, NY 10016 USA; 6 Division of Psychiatry and Applied Psychology, School of Medicine, University of Nottingham, Nottingham, NG72UH UK; 7 Institute for Genomic Statistics and Bioinformatics, University Hospital Bonn, Bonn, Germany; 8 Department of Psychiatry, University of Münster, Münster, Germany; 9 Department of Psychiatry, Melbourne Medical School, The University of Melbourne, Melbourne, VIC Australia; 10 The Florey Institute of Neuroscience and Medical Health, The University of Melbourne, Parkville, VIC Australia; 11 Departments of Psychiatry and of Neuroscience and Physiology, SUNY Upstate Medical University, Syracuse, 13210 NY USA; 12 K.G. Jebsen Centre for Research on Neuropsychiatric Disorders, University of Bergen, Bergen, Norway; 13 Biological Psychiatry Unit, IRCCS Istituto Centro San Giovanni di Dio Fatebenefratelli, Brescia, Italy

**Keywords:** Genetics, Neuroscience

## Abstract

The identification of biomarkers to support the diagnosis and prediction of treatment response for attention-deficit/hyperactivity disorder (ADHD) is still a challenge. Our previous works highlighted the *DRD4* (dopamine receptor D4) as the best potential genetic marker for childhood diagnosis and methylphenidate (MPH) response. Here, we aimed to provide additional evidence on biomarkers for ADHD diagnosis and treatment response, by using more specific approaches such as meta-analytic and bioinformatics tools. Via meta-analytic approaches including over 3000 cases and 16,000 controls, we demonstrated that, among the different variants studied in *DRD4* gene, the 48-base pair, Variable Tandem Repeat Polymorphism, VNTR in exon 3 showed an age/population-specificity and an allelic heterogeneity. In particular, the 7R/“long” allele was identified as an ADHD risk factor in European-Caucasian populations (*d* = 1.31, 95%CI: 1.17–1.47, *Z* = 4.70/*d* = 1.36, 95%CI: 1.20–1.55, *Z* = 4.78, respectively), also, from the results of last meta-analysis, linked to the poor MPH efficacy. The 4R/“short” allele was a protective factor in European-Caucasian and South American populations (*d* = 0.83, 95%CI: 0.75–0.92, *Z* = 3.58), and was also associated to positive MPH response. These results refer to children with ADHD. No evidence of such associations was detected for adults with persistent ADHD (data from the last meta-analysis). Moreover, we found evidence that the 4R allele leads to higher receptor expression and increased sensitivity to dopamine, as compared with the 7R allele (*d* = 1.20, 95%CI: 0.71–1.69, *Z* = 4.81), and this is consistent with the ADHD protection/susceptibility effects of the respective alleles. Using bioinformatics tools, based on the latest genome-wide association (GWAS) meta-analysis of the Psychiatry Genomic Consortium (PGC), we demonstrated that the 48 bp VNTR is not in Linkage Disequilibrium with the *DRD4* SNPs (Single Nucleotide Polymorphisms), which were not found to be associated with ADHD. Moreover, a *DRD4* expression downregulation was found in ADHD specific brain regions (Putamen, *Z* score = −3.02, *P* = 0.00252). Overall, our results suggest that *DRD4* 48 bp VNTR variants should be considered as biomarkers to support the diagnosis of ADHD and to predict MPH response, although the accuracy of such a biomarker remains to be further elucidated.

## Introduction

Attention-deficit/hyperactivity disorder (ADHD) is a complex neurodevelopmental disorder, characterized by age-inappropriate symptoms of inattention and/or hyperactivity-impulsivity, with a heterogeneous clinical phenotype^1^. The worldwide prevalence among school-aged children is around 5%^2^. About 65% of affected individuals continue to exhibit impairing ADHD symptoms into adulthood^3^. ADHD prevalence in adults is estimated at 2.5%^4^.

The severity level and presentation of ADHD changes over the lifespan, with adult patients displaying less obvious symptoms of hyperactivity and impulsivity^5^. Moreover, changes in structural brain abnormalities from childhood to adulthood with ADHD have been reported^6^, suggesting potential differential causes for the onset and persistence of the disorder^7^.

ADHD aetiology is not yet completely understood. Despite evidence that environmental factors (e.g., maternal smoking, low birth weight, and prematurity) play a significant role, genetic studies support a strong genetic contribution. Indeed, average heritability was estimated at 76%^8,9^, in childhood and at 30–50%^10–12^ or even greater^13,14^ in adulthood. The most recent and largest genome-wide association (GWAS) meta-analysis from the Psychiatric Genomics Consortium (PGC) identified common single-nucleotide (SNPs) variants, surpassing genome-wide significance in 12 independent loci^15^, providing important new insights into the neurobiology of childhood ADHD. Additional insight comes from the studies on the crucial role played by rare variants^9^.

Pharmacotherapy is a crucial component for the treatment of ADHD^16^. Taking into account both efficacy and safety, evidence from a recent network meta-analysis^17^ supports methylphenidate (MPH) in children and adolescents, and amphetamines, in adults, as possible first-choice medications for the short-term treatment of ADHD, suggesting once again potential neurobiological differences across the lifespan.

In the era of precision medicine, the biomarker approach to diagnosis and treatment offers the opportunity to improve diagnostic assessment and provides insights into etiological mechanisms. As it is known that a considerable proportion (35%) of ADHD patients do not respond to available first line medication, this approach has also the potential to contribute to individualized therapies. The DRD4 (dopamine receptor D4) is a G-protein-coupled receptor belonging to the D2-like receptor family, which modulates intracellular signalling by inhibiting the production of the second messenger cyclic AMP (cAMP) level^18,19^ and is responsible for neuronal signalling in the mesolimbic system of the brain. It is specifically involved in dopamine synthesis, release and neuronal firing^18^. It has been considered a candidate for the aetiology of ADHD due to its high expression in brain regions implicated in attention and inhibition, such as the orbitofrontal and anterior cingulate cortex^20,21^. Additional interest derived from a link with the personality trait of novelty seeking^22,23^, which has been compared with the high levels of impulsivity and excitability often seen in ADHD^24^. Further, the *DRD4* “knockout” mouse exhibits a heightened response to cocaine and methamphetamine relative to controls, as indicated by increases in loco-motor behaviour^25^. The *DRD4* gene comprises four exons and encodes a putative 387-amino acid protein with seven transmembrane domains, where the most widely studied 48 bp VNTR (variable tandem repeat) polymorphism encodes the third cytoplasmic loop. This multiallelic polymorphism includes 11 copies of a 48-bp repeat sequence, where the 4, 7 and 2 repeat (R) alleles are the most prevalent. Genetic demographic studies report that the 7R allele is present in highly varying percentages in different populations worldwide^26–30^. It is known that this polymorphism impacts on mRNA and protein expression levels, indicating a significant functional biological effect of this polymorphism on the translation of the respective protein^31^. After the exon 3 VNTR, the other *DRD4* polymorphisms studied are found in the promoter region of the gene: 120 bp duplication (rs4646984); −521 C/T (rs1800955), −616 C/G (rs747302); 12 bp (rs4646983), −615 A/G (rs936462), −376 C/T (rs916455).

In our previous works^7,32,33^, we strongly suggested that *DRD4* along with dopamine transporter gene (*SLC6A3*) are significant predictors of childhood ADHD susceptibility, different endophenotypes, MPH response, and linked to altered genes expression levels. However, the latest GWAS/meta-analysis^15^ did not detect associations with these “classical” candidate genes.

Here, we build on and expand our previous studies, focusing on *DRD4*, to further assess its role as a potential biomarker for the diagnosis of ADHD and for MPH response, both in children and adults. Up-date and new meta-analyses were performed to statistically assess the association with ADHD in childhood and to confirm the functional role of the 48 bp VNTR. Bioinformatics in silico analyses were conducted to understand the impact of *DRD4* gene and of 48 bp VNTR polymorphism in the pathology and to reconcile our positive findings with the negative results for five *DRD4* SNPs in the GWAS of Demontis et al.^15^. We used also bioinformatics tools to confirm the functional role of *DRD4* in specific ADHD brain regions. In addition, after the literature research on the association between *DRD4* polymorphisms and ADHD susceptibility in children with ADHD and MPH response in ADHD adulthood, we concluded that there are not enough studies to perform meta-analyses.

So far as the literature research does not add further studies to the meta-analytic approach, we reported the results from the last more recent meta-analyses, and this regards the associations of SNPs and ADHD susceptibility in children with ADHD, as well as the 48 bp/SNPs with ADHD susceptibility in adulthood and with MPH response in ADHD childhood and adulthood.

## Materials and methods

### Meta-analysis

#### DRD4 polymorphisms in children with ADHD

##### Search strategy and selection criteria

According to the PRISMA guidelines^34^, we searched the electronic databases PubMed, Embase and “ADHDgene Database” (http://adhd.psych.ac.cn/), up to December 2018, with no restrictions on language, date, or article type. In PubMed, we used the following search terms/syntax “ADHD OR attention deficit OR attention-deficit OR attention deficit hyperactivity disorder OR attention-deficit hyperactivity disorder OR hyperkinetic syndrome OR hyperkinetic disorder OR hyperactivity disorder OR hyperactive child syndrome” AND “children OR child” AND “*DRD4* OR dopamine receptor D4, AND “gene”, AND “polymorphisms”, AND “SNP OR Single Nucleotide polymorphism”, AND “VNTR OR variable tandem repeats”, AND “association”, AND “TDT OR Transmission Disequilibrium Test, OR family-based” AND “methylphenidate OR MPH”, AND “pharmacogenetics”, AND “drugs”, AND “treatments”, AND “clinical trials” AND “meta-analy* OR metaanaly*”. During the research, we identified different meta-analyses, however we took in consideration those more recent: Gizer and colleagues^35^, Wu and colleagues^36^; Nikolaidis and Gray^37^; Myer and colleagues^38^, to cross-check their references to find any publications possibly missed in our electronic search. The literature search was performed independently by two individuals (CS, CB). Disagreements were resolved by the other authors.

The Newcastle-Ottawa Scale was used to assess quality of studies^39^.

##### Inclusion and exclusion criteria

We selected articles that met the following inclusion criteria: ADHD diagnosis according to the Diagnostic and Statistical Manual of Mental Disorders (DSM-III, DSM-III-R, DSM-IV, DSM-IV-TR) or equivalent Hyperkinetic disorder or the International Classification of Diseases 10th Revision (ICD-10) or previous versions; case–control and a family-based study design for genetic studies; clinical trials for pharmacogenetic studies. We excluded studies (a) using comparisons with a family control (healthy siblings, to avoid the deviation from Hardy-Weinberg Equilibrium); (b) using samples fully overlapping with other included studies; (c) for which data to perform analyses were not available, even after contacting the study corresponding authors.

##### Data extraction for meta-analyses

CS and CB independently extracted the following data: first author, study design, year of publication, populations studied, study design, sample size, ethnic groups, and key results from each study.

##### Statistical analyses

Review Manager was used to perform the meta-analysis (RevMan Version 5.1.6; Copenhagen, The Nordic Cochrane Centre, The Cochrane Collaboration, 2008). We used the random-effects model to generate a pooled effect size and 95% confidence interval (CI) from individual study effect sizes (the odd ratios for genetics studies using the Mantel–Haenszel, M-H). The significance of the pooled effect sizes was determined by *z*-tests. Between-study heterogeneity was assessed using a *χ*^2^ test of goodness of fit test and the *I*^2^ statistic. We used a *P*-value < 0.05 to indicate statistical significance.

Publication bias was estimated using the method by Egger and colleagues^40^ which relies on a linear regression approach to measure funnel plot asymmetry on the natural logarithm scale of the effect size. The significance of the intercept (a) was determined by the *t*-test^40^. The rank correlation method and regression method tests were conducted using MIX version 1.7 (http://www.mix-for-meta-analysis.info).

In relation to 48 bp multiallelic variants, the meta-analyses were conducted comparing 7R versus others, 4R allele versus others and 2R alleles versus others. Based on different pharmacological characteristics^22,31^, we divided these repeat alleles also into “short” (two to four) and “long” (five to eight)^41–43^ and conducted the meta-analyses considering “long” allele versus others.

#### DRD4 polymorphisms in adults with ADHD

Search strategy and selection criteria, inclusion and exclusion criteria, and statistical analyses were conducted as above, except for the term “adults” instead of “children OR child”. During the research, we identified the most recent meta-analysis^44^, and we reported their findings, because no additional studies have been performed.

#### Focus on DRD4 48 bp VNTRs polymorphism: functional differences

We cross checked the references of the latest review describing the different studies on the functional biological effect of the 48 bp VNTR polymorphism^45^ to find any publications possibly missed in our electronic search and did an updated search through to December 2018. We performed meta-analyses for 2R allele versus 4R, 2R versus 7R and 4R versus 7R. Statistical analyses were conducted as above.

### Bioinformatics in silico analyses

From 1000 Genome database in which the five SNPs found negative in last GWAS^15^ (rs752306, rs7124601, rs146876215, rs1870723, rs7482904) are included, we built a population specific-linkage disequilibrium (LD) block by using Haploview software.

With the aim to further investigate the involvement of *DRD4* on ADHD aetiology we performed a Transcription Wide Association Study (TWAS) considering the last available summary statistics for ADHD in the PGC portal (https://www.med.unc.edu/pgc/). TWAS is a gene association method estimating whether a different gene expression regulation (e.g., up or downregulation) could be expected for the analysed phenotype based on GWAS associations. This can be done through the imputation of the genetic component of gene expression using tissue-specific cis-eQTL models^46^. In our analysis, we considered cis-eQTL models (http://predictdb.org/) trained on the Genotype-Tissue Expression database, i.e., GTEx (https://gtexportal.org/home/) and we specifically focus on brain tissues.

## Results

### Meta-analysis

#### DRD4 polymorphisms in children with ADHD

##### 48 bp VNTR polymorphism

The PRISMA flow chart is in Supplementary Fig. S1. After screening 154 records, we selected 77 studies meeting our eligibility criteria: 43 studies case–control (CC), 21 family-based studies (TDT, transmission disequilibrium test) and 13 (combined case–control and transmission disequilibrium test approaches). Results in relation to different populations (Asian, European-Caucasian, Middle Eastern and South American) are reported in Table 1.

**Table 1:** List of studies included in the meta-analyses of the 48 bp VNTR in *DRD4* gene.

Authors [Reference]	Case–control; TDT	Years	Populations	ADHD	Controls	Families	Results	Ethnic grouping	Caucasian	Hispanic	African-american	Asian	others
Qian Q [1]	cc-tdt	2004	China	307	165	160	No/no	Asian				Chinese	
Leung PW [2]	cc	2005	China	32	247		Yes	Asian				Chinese	
Cheuk DK [3]	cc-tdt	2006	China	64	64	64	Trend/no	Asian				Chinese	
Leung PW [4]	tdt	2017	China			33	Yes	Asian				Chinese	
Bhaduri N [5]	cc-tdt	2006	India	50	50		No	Asian				Indian	
Das M [6]	cc-tdt	2011	India	126	96	123	No/no	Asian					Indo-caucasoid
Maitra S [7]	cc	2014	India	160	120		No	Asian					Indo-caucasoid
Stanley A [8]	cc	2017	India	44	44		No	Asian				Indian	
Kim YS [9]	tdt	2005	Korea			126	No	Asian				Korean	
Cho SC [10]	cc-tdt	2007	Korea	116	133		No/no	Asian				Korean	
Ji HS [11]	cc	2012	Korea	114	84		No	Asian				Korean	
Kim H [12]	cc	2017	Korea	255	98		No	Asian				Korean	
Kim JI [13]	cc	2018	Korea	67	44		No	Asian				Korean	
Hong JH [14]	cc	2018	Korea	150	322		Yes	Asian				Korean	
Brookes KJ [15]	tdt	2005	Taiwan			198	No	Asian				Taiwanese	
LaHoste GJ [16]	cc	1996	Canada	39	39		Yes	European-Caucasian	85%	12.50%	2.50%		
Perkovic MN [17]	cc	2014	Croatia	102	128		Yes	European-Caucasian	Caucasian				
Bakker SC [18]	tdt	2005	Dutch			236	No	European-Caucasian	Caucasian				
Altink ME [19]	cc	2012	Dutch (IMAGE)	350	195		No	European-Caucasian	Caucasian				
El-Faddagh M [20]	cc	2004	Germany	24	102		Yes	European-Caucasian	Caucasian				
Becker K [21]	cc	2010	Germany	63	237		No	European-Caucasian	Caucasian				
Niederhofer H [22]	tdt	2008	Germany, Austria			36	No	European-Caucasian	Caucasian				
Albrecht B [23]	cc	2014	Germany, Switzerland	94	31		No	European-Caucasian	Caucasian				
Kereszturi E [24]	cc-tdt	2008	Hungary	173	284		No	European-Caucasian	Caucasian				
Sonuga-Barke EJS [25]	cc	2008	IMAGE	702	694		No	European-Caucasian	Caucasian				
Hawi Z [26]	cc-tdt	2000	Ireland	99	88	78	No/no	European-Caucasian	Caucasian				
Kirley A [27]	tdt	2002	Ireland			118	No	European-Caucasian	Caucasian				
Lowe N [28]	tdt	2004	Ireland			178	No	European-Caucasian	Caucasian				
Johnson KA [29]	cc	2008	Ireland	68	60		No	European-Caucasian	Caucasian				
Gomez-Sanchez CI [30]	cc	2016	Spain	289	338		No	European-Caucasian	Caucasian				
Holmes J [31]	cc-tdt	2000	UK	129	442	133	Yes/yes	European-Caucasian	Caucasian				
Mill J [32]	cc-tdt	2001	UK	264	378	85	Yes/yes	European-Caucasian	Caucasian				
Curran S [33]	cc	2001	UK	133	91		Yes	European-Caucasian	Caucasian				
Payton A [34]	cc	2001	UK	50	42		No	European-Caucasian	Caucasian				
Holmes J [35]	tdt	2002	UK			51	Yes	European-Caucasian	Caucasian				
Paloyelis Y [36]	cc	2010	UK (IMAGE)	36	31		No	European-Caucasian	Caucasian				
Mill J [32]	cc	2006	UK, New Zeland				Yes	European-Caucasian	Caucasian				Dunedin (New Zealand)
Faraone SV [37]	tdt	1999	USA			27	Yes	European-Caucasian					
Comings DE [38]	cc	1999	USA	52	368		Yes	European-Caucasian	white non-Hispanic				
Barr CL [39]	tdt	2000	USA			82	Yes	European-Caucasian					
Lunetta KL [40]	tdt	2000	USA			44	Yes	European-Caucasian					
McCracken JT [41]	tdt	2000	USA			197	Yes	European-Caucasian	81%				
Todd RD [42]	tdt	2001	USA			201	No	European-Caucasian					
Maher BS [43]	tdt	2002	USA			33	No	European-Caucasian	71.5%	27.7%	19.5%	4.10%	3.5% Native American
Smith KM [44]	cc	2003	USA	158	81		No	European-Caucasian	94%	1%	5%		
Kustanovich V [45]	tdt	2004	USA			293	Yes	European-Caucasian	79%	4%	2%	2%	13%
Gornick MC [46]	cc-tdt	2007	USA	166	282	113	Yes/yes	European-Caucasian	75%	10%	12%	2%	1%
Shaw P [47]	cc	2007	USA	105	103		Yes	European-Caucasian	75%	10%	13%	0%	2%
Lee SS & Humphreys KL [48]	cc	2014	USA	119	110		No	European-Caucasian	49%	9%	8%	3%	22% mixed, 10% others
Rowe DC [49]	cc	1998	USA, Atlanta	107	58		Yes	European-Caucasian	71.80%	4.30%	8.50%		
Swanson JM [50]	tdt	1998	USA, California, Irvine			52	Yes	European-Caucasian	79.70%	11.40%	3.60%	2.80%	2% native american, 0.4% pacific island
Grady DL [51]	cc	2003	USA, California, Irvine	132	1652		Yes	European-Caucasian	79.70%	11.40%	3.60%	2.80%	2% native american, 0.4% pacific island
Sunohara GA [52]	tdt	2000	USA, California, Irvine; Canada, Toronto			199	Yes	European-Caucasian					
Smalley SL [53]	tdt	1998	USA, California, Los Angeles			133	Yes	European-Caucasian	80%				
Bidwell LC [54]	cc	2011	USA, Colorado	202	93		Yes	European-Caucasian					
Reiersen AM and Todorov AA [55]	cc	2011	USA, Missouri	142	812		Yes	European-Caucasian	Caucasian				
Frank Y [56]	cc	2004	USA, New York	81	24		No	European-Caucasian					
Castellanos FX [57]	cc	1998	USA, Washington	82	112		No	European-Caucasian	white non-Hispanic				
Shahin O [58]	cc	2015	Egypt	29	31		Yes	Middle Eastern					Egyptian
ElBaz Mohamed F [59]	cc	2017	Egypt	50	50		Yes	Middle Eastern					Egyptian
Tabatabaei SM [60]	cc	2017	Iran	130	130		Yes	Middle Eastern	Caucasian				Turkish
Eisenberg J [61]	tdt	2000	Israel			46	No	Middle Eastern					Ashkenazi-non Ashkenazy
Kotler M [62]	cc	2000	Israel	49	49		Yes	Middle Eastern					Ashkenazi-non Ashkenazy
Manor I [63]	cc-tdt	2002	Israel	360	1908	178	Trend/yes	Middle Eastern					Ashkenazi-non Ashkenazy
Tahir E [64]	tdt	2000	Turkey			26	Yes	Middle Eastern					Turkish
Guney E [65]	cc	2013	Turkey	50	50		No	Middle Eastern					Turkish
Ercan ES [66]	cc	2016	Turkey	201	100		No	Middle Eastern					Turkish
Akay AP [67]	cc	2018	Turkey	20	50		No	Middle Eastern					Turkish
Roman T [68]	cc-tdt	2001	Brazil	132	200	77	Yes/yes	South American	Caucasian				African or Native American admixture
Tovo-Rodrigues L [69]	cc	2012	Brazil	66	37		No	South American	Caucasian				African or Native American admixture
Tovo-Rodrigues L [70]	cc	2013	Brazil	339	2926		No	South American	Caucasian				African or Native American admixture
Carrasco X [71]	cc	2004	Chile	26	25		Yes	South American	70%				30% Amerindian
Carrasco X [72]	cc	2006	Chile	26	25		Yes	South American	70%				30% Amerindian
Henriquez-Henriquez M [73]	cc	2012	Chile	20	20		No	South American	70%				30% Amerindian
Arcos-Burgos M [74]	cc-tdt	2004	Colombia	99	94	56	No/no	South American					Paisa Antioquia community genetic isolate
Fonseca DJ [75]	tdt	2015	Colombia			86	No	South American					
Martinez-Levy G [76]	cc	2009	Mexico	105	84		No	South American					

We structured this paragraph reporting the results in relation to (a) the comparisons using as dependent variable the allele comparison (allele 2R versus others; allele 4R versus others; allele 7R versus others; long allele versus others); (b) merged data between the two genetic approaches: CC and TDT studies for alleles 2R, 4R, 7R; (c) publication bias and (d) Newcastle-Ottawa Scale.

##### ***Allele 2R versus others***

The results are showed in Supplementary Fig. S2 and summarized in Table 2.

**Table 2:** Summary of the results obtained after meta-analyses.

	Case/trasmitted		Control/untrasmitted				
	Events	Total events	Events	Total events	Odd ratio, M-H, Random, 95% CI	Heterogeneity	Test for overall effect
*Allele 2*							
Asian							
CC	391	2632	438	2674	0.96 [0.73, 1.27]	Tau² = 0.12; Chi² = 25.00, df = 10 (*P* = 0.005); *I*² = 60%	*Z* = 0.27 (*P* = 0.79)
TDT	172	849	171	849	1.01 [0.79, 1.28]	Tau² = 0.00; Chi² = 3.96, df = 5 (*P* = 0.56); *I*² = 0%	*Z* = 0.04 (*P* = 0.96)
European-Caucasian							
CC	288	3366	550	6094	1.07 [0.85, 1.33]	Tau² = 0.07; Chi² = 23.08, df = 14 (*P* = 0.06); *I*² = 39%	*Z* = 0.56 (*P* = 0.57)
TDT	220	1890	248	1889	0.87 [0.71, 1.06]	Tau² = 0.00; Chi² = 10.64, df = 11 (*P* = 0.47); *I*² = 0%	*Z* = 1.40 (*P* = 0.16)
Middle Eastern							
CC	55	616	33	620	1.95 [0.37, 10.29]	Tau² = 2.72; Chi² = 25.05, df = 4 (*P* < 0.0001); *I*² = 84%	*Z* = 0.79 (*P* = 0.43)
TDT	7	66	6	64	1.15 [0.36, 3.62]	Not applicable	*Z* = 0.23 (*P* = 0.82)
South American							
CC	96	1254	514	6522	1.15 [0.73, 1.80]	Tau² = 0.10; Chi² = 5.83, df = 3 (*P* = 0.12); *I*² = 49%	*Z* = 0.61 (*P* = 0.54)
CC	4	56	2	56	2.08 [0.36, 11.83]	Not applicable	*Z* = 0.82 (*P* = 0.41)
*Allele 4*							
Asian							
CC	2087	2632	2094	2674	1.00 [0.83, 1.21]	Tau² = 0.03; Chi² = 15.60, df = 10 (*P* = 0.11); *I*² = 36%	*Z* = 0.04 (*P* = 0.97)
TDT	686	950	599	950	1.85 [0.94, 3.63]	Tau² = 0.73; Chi² = 58.64, df = 6 (*P* < 0.00001); *I*² = 90%	*Z* = 1.78 (*P* = 0.07)
European-Caucasian							
CC	2143	3366	4196	6094	0.79 [0.69, 0.91]	Tau² = 0.03; Chi² = 26.67, df = 14 (*P* = 0.02); *I*² = 48%	*Z* = 3.31 (*P* = 0.0009)
TDT	1020	1890	1054	1889	0.89 [0.73, 1.10]	Tau² = 0.07; Chi² = 24.33, df = 11 (*P* = 0.01); *I*² = 55%	*Z* = 1.08 (*P* = 0.28)
Middle Eastern							
CC	428	684	406	720	1.14 [0.49, 2.66]	Tau² = 0.86; Chi² = 39.51, df = 5 (*P* < 0.00001); *I*² = 87%	*Z* = 0.31 (*P* = 0.76)
TDT	32	66	27	64	1.29 [0.65, 2.58]	Not applicable	*Z* = 0.72 (*P* = 0.47)
South American							
CC	848	1426	4148	6636	0.82 [0.65, 1.04]	Tau² = 0.04; Chi² = 9.91, df = 5 (*P* = 0.08); *I*² = 50%	*Z* = 1.66 (*P* = 0.10)
TDT	41	56	41	56	1.00 [0.43, 2.31]	Not applicable	*Z* = 0.00 (*P* = 1.00)
*Allele 7*							
Asian							
CC	13	1789	18	2176	0.84 [0.39, 1.80]	Tau² = 0.00; Chi² = 4.90, df = 8 (*P* = 0.77); *I*² = 0%	*Z* = 0.46 (*P* = 0.65)
TDT	5	265	4	265	1.27 [0.33, 4.87]	Tau² = 0.00; Chi² = 1.02, df = 2 (*P* = 0.60); *I*² = 0%	*Z* = 0.35 (*P* = 0.72)
European-Caucasian							
CC	2020	7618	4279	16506	1.25 [1.07, 1.45]	Tau² = 0.11; Chi² = 104.24, df = 26 (*P* < 0.00001); *I*² = 75%	*Z* = 2.77 (*P* = 0.006)
TDT	916	3202	720	3201	1.40 [1.23, 1.59]	Tau² = 0.01; Chi² = 23.89, df = 20 (*P* = 0.25); *I*² = 16%	*Z* = 5.09 (*P* < 0.00001)
Middle Eastern							
CC	92	986	124	820	0.61 [0.45, 0.83]	Tau² = 0.00; Chi² = 4.38, df = 5 (*P* = 0.50); *I*² = 0%	*Z* = 3.13 (*P* = 0.002)
TDT	35	164	28	162	1.34 [0.54, 3.31]	Tau² = 0.26; Chi² = 2.54, df = 1 (*P* = 0.11); *I*² = 61%	*Z* = 0.63 (*P* = 0.53)
South American							
CC	393	1490	1321	6696	1.25 [0.95, 1.65]	Tau² = 0.07; Chi² = 14.05, df = 6 (*P* = 0.03); *I*² = 57%	*Z* = 1.59 (*P* = 0.11)
TDT	59	313	58	313	1.02 [0.68, 1.53]	Tau² = 0.00; Chi² = 0.86, df = 2 (*P* = 0.65); *I*² = 0%	*Z* = 0.10 (*P* = 0.92)
*Long allele*							
Asian							
CC	72	2952	61	2914	1.22 [0.83, 1.78]	Tau² = 0.01; Chi² = 11.15, df = 11 (*P* = 0.43); *I*² = 1%	*Z* = 1.01 (*P* = 0.31)
TDT	32	679	20	679	1.49 [0.65, 3.44]	Tau² = 0.32; Chi² = 6.17, df = 4 (*P* = 0.19); *I*² = 35%	*Z* = 0.94 (*P* = 0.35)
European-Caucasian							
CC	848	3560	1072	6311	1.41 [1.19, 1.67]	Tau² = 0.06; Chi² = 32.56, df = 15 (*P* = 0.005); *I*² = 54%	*Z* = 4.04 (*P* < 0.0001)
TDT	531	1869	448	1864	1.28 [1.05, 1.56]	Tau² = 0.04; Chi² = 17.36, df = 11 (*P* = 0.10); *I*² = 37%	*Z* = 2.49 (*P* = 0.01)
Middle Eastern							
CC	133	976	515	2588	0.62 [0.41, 0.93]	Tau² = 0.13; Chi² = 11.37, df = 5 (*P* = 0.04); *I*² = 56%	*Z* = 2.32 (*P* = 0.02)
TDT	64	181	90	179	0.63 [0.19, 2.06]	Tau² = 0.62; Chi² = 6.58, df = 1 (*P* = 0.01); *I*² = 85%	*Z* = 0.76 (*P* = 0.45)
South American							
CC	295	1157	1267	5864	1.13 [0.90, 1.43]	Tau² = 0.02; Chi² = 4.82, df = 3 (*P* = 0.19); *I*² = 38%	*Z* = 1.05 (*P* = 0.29)
TDT	11	56	9	56	1.28 [0.48, 3.37]	Not applicable	*Z* = 0.49 (*P* = 0.62)

*In Asian populations*: (a) CC: Random model *Z* = 0.27, *P* = 0.79, in presence of heterogeneity in effect size across the studies: *P* = 0.005, *I*^2^ = 60%; (b) TDT: Random model *Z* = 0.04, *P* = 0.96, in absence of heterogeneity in effect size across the studies: *P* = 0.56, *I*^2^ = 0%.

*In European-Caucasian populations*: (a) CC: Random model *Z* = 0.56, *P* = 0.57, without heterogeneity in effect size across the studies: *P* = 0.06, *I*^2^ = 39%; (b) TDT: Random model *Z* = 1.40, *P* = 0.16, without heterogeneity in effect size across the studies: *P* = 0.47, *I*^2^ = 0%.

*In Middle Eastern populations*: (a) CC: Random model *Z* = 0.79, *P* = 0.43, with heterogeneity in effect size across the studies: *P* < 0.0001, *I*^2^ = 84%; (b) TDT: Random model *Z* = 0.23, *P* = 0.82.

*In South American populations*: (a) CC: Random model *Z* = 0.61, *P* = 0.54, without heterogeneity in effect size across the studies: *P* = 0.12, *I*^2^ = 49%; (b) TDT: Random model *Z* = 0.82, *P* = 0.41.

##### ***Allele 4R versus others***

The results are showed in Supplementary Fig. S3 and summarized in Table 2.

*In Asian populations*: (a) CC: Random model *Z* = 0.04, *P* = 0.97, without heterogeneity in effect size across the studies *P* = 0.11, *I*^2^ = 36%; (b) TDT: Random model *Z* = 1.78, *P* = 0.07, with heterogeneity in effect size across the studies *P* < 0.00001, *I*^2^ = 90%.

*In European-Caucasian populations*: (a) CC: Random model *Z* = 3.31, *P* = 0.0009, d = 0.79 95%CI: 0.69–0.91, with slightly heterogeneity in effect size across the studies *P* = 0.02, *I*^2^ = 48%; (b) TDT: Random model *Z* = 1.08, *P* = 0.28, with slightly heterogeneity in effect size across the studies *P* = 0.01, *I*^2^ = 55%.

*In Middle Eastern populations*: (a) CC: Random model *Z* = 0.31, *P* = 0.76, with heterogeneity in effect size across the studies *P* < 0.00001, *I*^2^ = 87%; (b) TDT: Random model *Z* = 0.72, *P* = 0.47.

*In South American populations*: (a) CC Random model *Z* = 1.66, *P* = 0.10, with no heterogeneity in effect size across the studies *P* = 0.08, *I*^2^ = 50%, (b) TDT: Random model *Z* = 0.00, *P* = 1.00.

##### ***Allele 7R versus others***

The results are showed in Supplementary Fig. S4 and summarized in Table 2.

*In Asian populations*: (a) CC: Random model *Z* = 0.46, *P* = 0.65, without heterogeneity in effect size across the studies *P* = 0.77, *I*^2^ = 0%; (b) TDT: Random model *Z* = 0.35, *P* = 0.72, without heterogeneity in effect size across the studies *P* = 0.60, *I*^2^ = 0%.

*In European-Caucasian populations*: (a) CC: Random model *Z* = 2.77, *P* = 0.006, *d* = 1.25 95%CI: 1.07–1.45, with heterogeneity in effect size across the studies *P* < 0.00001, *I*^2^ = 75%; (b) TDT Random model *Z* = 5.09, *P* < 0.00001, *d* = 1.40 95%CI: 1.23–1.59 in absence of heterogeneity in effect size across the studies *P* = 0.25, *I*^2^ = 16%.

*In Middle Eastern populations:* (a) CC: Random model *Z* = 3.13, *P* = 0.002, *d* = 0.61 95%CI: 0.45–0.83 in absence of heterogeneity in effect size across the studies *P* = 0.50, *I*^2^ = 0%; (b) TDT: Random model *Z* = 0.63, *P* = 0.53, in absence of heterogeneity in effect size across the studies *P* = 0.11, *I*^2^ = 61%.

*In South American populations*: (a) CC: Random model *Z* = 1.59, *P* = 0.11, with a trend in heterogeneity in effect size across the studies *P* = 0.03, *I*^2^ = 57%; (b) TDT: Random model *Z* = 0.10, *P* = 0.92, in absence of heterogeneity in effect size across the studies *P* = 0.65, *I*^2^ = 0%.

##### ***Long allele versus others***

The results are showed in Supplementary Fig. S5 and summarized in Table 2.

*In Asian populations*: (a) CC: Random model *Z* = 1.01, *P* = 0.31, in absence of heterogeneity in effect size across the studies *P* = 0.43, *I*^2^ = 1%, (b) TDT: Random model *Z* = 0.94, *P* = 0.35, in absence of heterogeneity in effect size across the studies *P* = 0.19, *I*^2^ = 35%.

*In European populations:* (a) CC: Random model *Z* = 4.04, *P* < 0.0001, *d* = 1.41 95%CI: 1.19–1.67, in presence of heterogeneity in effect size across the studies *P* = 0.005, *I*^2^ = 54%, (b) TDT: Random model *Z* = 2.49, *P* = 0.01, *d* = 1.28 95%CI: 1.05–1.56, in absence of heterogeneity in effect size across the studies *P* = 0.10, *I*^2^ = 37%.

*In Middle Eastern populations:* (a) CC: Random model *Z* = 2.32, *P* = 0.02, *d* = 0.62 95%CI: 0.41–0.93, with a trend of heterogeneity in effect size across the studies *P* = 0.04, *I*^2^ = 56%, (b) TDT: Random model *Z* = 0.76, *P* = 0.45, with heterogeneity in effect size across the studies *P* = 0.01, *I*^2^ = 85%.

*In South American populations*: (a) CC: Random model *Z* = 1.05, *P* = 0.29, in absence of heterogeneity in effect size across the studies *P* = 0.19, *I*^2^ = 38%, (b) TDT: Random model *Z* = 0.49, *P* = 0.62.

##### ***Merged data between the two approaches CC and TDT for alleles 2R, 4R, 7R***

Table 3 shows the merged data from the CC and TDT studies.

**Table 3:** Summary results when meta-analyses performed in case–control studies are united with those performed in transmission disequilibrium test (TDT) for each allele of the 48 bp VNTR in *DRD4* gene.

	Case/trasmitted	Control/untrasmitted					
Allele	Events	Total events	Events	Total events	Odd ratio, M-H, Random, 95% CI	Heterogeneity	Test for overall effect
2							
Asian	563	3481	609	3523	0.98 [0.81, 1.19]	Tau² = 0.07; Chi² = 29.16, df = 16 (*P* = 0.02); *I*² = 45%	*Z* = 0.23 (*P* = 0.82)
European-Caucasian	508	5256	798	7983	0.98 [0.84, 1.14]	Tau² = 0.04; Chi² = 35.69, df = 26 (*P* = 0.10); *I*² = 27%	*Z* = 0.29 (*P* = 0.77)
Middle Eastern	62	682	39	684	1.60 [0.45, 5.73]	Tau² = 1.81; Chi² = 24.56, df = 5 (*P* = 0.0002); *I*² = 80%	*Z* = 0.72 (*P* = 0.47)
South American	100	1310	516	6578	1.18 [0.79, 1.78]	Tau² = 0.08; Chi² = 6.36, df = 4 (*P* = 0.17); *I*² = 37%	*Z* = 0.80 (*P* = 0.42)
4							
Asian	2773	3582	2693	3624	1.25 [0.95, 1.64]	Tau² = 0.26; Chi² = 83.77, df = 17 (*P* < 0.00001); *I*² = 80%	*Z* = 1.59 (*P* = 0.11)
European-Caucasian	3163	5256	5250	7983	0.83 [0.74, 0.94]	Tau² = 0.05; Chi² = 54.33, df = 26 (*P* = 0.0009); *I*² = 52%	*Z* = 3.08 (*P* = 0.002)
Middle Eastern	460	750	433	784	1.15 [0.57, 2.33]	Tau² = 0.68; Chi² = 39.64, df = 6 (*P* < 0.00001); *I*² = 85%	*Z* = 0.39 (*P* = 0.69)
South American	889	1482	4189	6692	0.83 [0.67, 1.03]	Tau² = 0.03; Chi² = 10.02, df = 6 (*P* = 0.12); *I*² = 40%	*Z* = 1.69 (*P* = 0.09)
European-Caucasian and South American	4052	6738	9439	14,675	0.83 [0.75, 0.92]	Tau² = 0.04; Chi² = 64.55, df = 33 (*P* = 0.0008); *I*² = 49%	*Z* = 3.58 (*P* = 0.0003)
7							
Asian	18	2054	22	2441	0.93 [0.48, 1.80]	Tau² = 0.00; Chi² = 6.18, df = 11 (*P* = 0.86); *I*² = 0%	*Z* = 0.22 (*P* = 0.82)
European-Caucasian	2936	10,820	4999	19,707	1.31 [1.17, 1.47]	Tau² = 0.09; Chi² = 138.89, df = 47 (*P* < 0.00001); *I*² = 66%	*Z* = 4.70 (*P* < 0.00001)
European-Caucasian without Sonuga-Barke et al.^47^	2476	9416	4475	18,319	1.33 [1.19, 1.49]	Tau² = 0.08; Chi² = 111.59, df = 46 (*P* < 0.00001); *I*² = 59%	*Z* = 5.13 (*P* < 0.00001)
European-Caucasian without Sonuga-Barke et al.^47^ and Altink et al.^48^	2276	8776	4333	17,963	1.36 [1.22, 1.50]	Tau² = 0.06; Chi² = 90.82, df = 45 (*P* < 0.0001); *I*² = 50%	*Z* = 5.82 (*P* < 0.00001)
Middle Eastern	127	1150	152	982	0.73 [0.50, 1.06]	Tau² = 0.11; Chi² = 12.02, df = 7 (*P* = 0.10); *I*² = 42%	*Z* = 1.65 (*P* = 0.10)
South American	452	1803	1379	7009	1.18 [0.95, 1.47]	Tau² = 0.04; Chi² = 15.15, df = 9 (*P* = 0.09); *I*² = 41%	*Z* = 1.53 (*P* = 0.13)
Long							
Asian	104	3631	81	3593	1.27 [0.89, 1.82]	Tau² = 0.05; Chi² = 17.74, df = 16 (*P* = 0.34); *I*² = 10%	*Z* = 1.33 (*P* = 0.18)
European-Caucasian	1379	5429	1520	8175	1.36 [1.20, 1.55]	Tau² = 0.05; Chi² = 51.33, df = 27 (*P* = 0.003); *I*² = 47%	*Z* = 4.78 (*P* < 0.00001)
Middle Eastern	197	1157	605	2767	0.61 [0.42, 0.88]	Tau² = 0.16; Chi² = 18.70, df = 7 (*P* = 0.009); *I*² = 63%	*Z* = 2.61 (*P* = 0.009)
South American	306	1213	1276	5920	1.13 [0.93, 1.38]	Tau² = 0.01; Chi² = 4.90, df = 4 (*P* = 0.30); *I*² = 18%	*Z* = 1.23 (*P* = 0.22)

The association with ADHD susceptibility was confirmed for allele 4R in European-Caucasian populations (Random model *Z* = 3.08, *P* = 0.002, *d* = 0.83 95%CI: 0.74–0.94, in presence of heterogeneity in effect size across the studies *P* = 0.0009, *I*^2^ = 52%). The statistical power increased when we combined the European-Caucasian with South American populations (Random model *Z* = 3.58, *P* = 0.0003, *d* = 0.83 95%CI: 0.75–0.92 in presence of heterogeneity in effect size across the studies *P* = 0.0008, *I*^2^ = 49%). Allele 7R was found associated in the European-Caucasian populations (Random model *Z* = 4.70, *P* < 0.00001, *d* = 1.31 95%CI: 1.17–1.47, in presence of heterogeneity in effect size across the studies *P* < 0.00001, *I*^2^ = 66%).

Concerning the results for the “long” allele, we found associations with ADHD susceptibility in European-Caucasian populations (Random model *Z* = 4.78, *P* < 0.00001, *d* = 1.36 95%CI: 1.20–1.55, in presence of heterogeneity in effect size across the studies *P* = 0.003, *I*^2^ = 47%), but with a protective effect in Middle Eastern population (Random model *Z* = 2.61, *P* = 0.009, *d* = 0.61 95%CI: 0.42–0.88, in presence of heterogeneity in effect size across the studies *P* = 0.009, *I*^2^ = 63%).

##### ***Publication bias***

The results of Egger’s test for publication bias are reported in Supplementary Table S1. Publication bias was found for studies of the 7R allele, mainly in the European-Caucasian populations (*P* = 0.018), with higher values when the CC and TDT findings were combined (*P* = 0.0004). Of note, we observed that, when we eliminated from the analyses the manuscripts from Sonuga-Barke and colleagues^47^ (*P* = 0.02) along with Altink and colleagues^48^ (*P* = 0.08), the values are less significant and the *P* value for the total sample was 0.83.

Analyses of the “long” and 4R alleles showed no publication bias.

##### ***Newcastle-Ottawa Scale***

In Supplementary Table S2, we reported the results of the Newcastle-Ottawa Scale for this polymorphism.

##### SNPs

Besides the VNTR, several SNPs were investigated. Our research did not add any other studies reported in the last meta-analysis by Wu and colleagues^36^. Thus, the results did not change for the 120 bp duplication (rs4646984); −521 (C/T) (rs1800955); −616 (C/G) (rs747302), 12 bp (rs4646983); −615 (A/G) (rs936462); −376 (C/T) (rs916455), that did not show significant results.

For other SNPs: rs7395429, rs3758653, rs11246228, rs752306^49–51^; rs4646984^52^; rs916457^53^; rs936465^54^, no-meta-analyses can be performed, because very few studies were available (minimum three studies), considering that Yu and colleagues^49,50^ and Chang and colleagues^51^ studied the same population.

#### DRD4 polymorphisms in MPH pharmacogenetic studies in children with ADHD

Regarding to the research on the MPH pharmacogenetic studies, we ascertained that no other new studies were published on this topic as compared with the last meta-analysis by Myer and colleagues^38^ on 48 bp VNTR. Thus, we reported their results and their analyses. In particular, the homozygous 4R genotype demonstrated an association with improved MPH response, when compared with other genotypes (OR: 1.66, 95%CI: 1.16–2.37, *P* = 0.005), whereas the meta-analysis of the 7R repeat allele versus others showed a trend with an OR = 0.68 (95%CI: 0.47–1.00, *P* = 0.05)^38^.

#### DRD4 polymorphisms in adults with ADHD

From the last meta-analysis^44^, no other studies on the topic were available to add to the analyses. Concerning 48 bp VNTR, no association was observed. Contrasting results have been reported for the 120 bp duplication (rs4646984) and negative results for rs3758653, and rs936465. In relation to those retrieved in the most recent meta-analyses^7,44^, no other additional studies were found.

#### DRD4 polymorphisms in MPH pharmacogenetic studies in adults with ADHD

Concerning 48 bp VNTR, two studies were available with negative results and one study on 120 bp duplication^44^.

#### Focus on 48 bp VNTR in DRD4 gene: functional differences

The last review by Pappa and colleagues^45^, that resumed the studies on the potential biological differences among *DRD4* VNTR variants, was updated and, because no other new studies were conducted since 2014 to date, we conducted meta-analysis on the papers reported in Pappa and colleagues^45^. The studies are divided according to in vitro, in vivo and in silico methodologies. There were enough studies (minimum three studies) to perform meta-analyses only for in vitro studies and they were divided according to technologies used: [^3^H]spiperone binding RIA; [^3^H]spiperone Ca^2+^ channel flux assay; [^35^S]GTPγS agonist stimulated binding assay; BRET_50_ assay; luciferase reporter assay; western analysis; transient transfection. In Table 4, we reported these studies along with the techniques, functional response, the cell cultures used and the agonists. In Supplementary Figs. S6, S7, S8, the meta-analyses report the association of the functionality of allele 2R versus 4R (Random model *Z* = 4.52; *P* < 0.00001, *d* = 0.86 95%CI: 0.48–1.23); allele 2R versus 7R (Random model *Z* = 4.54; *P* < 0.00001, *d* = 1.07 95%CI: 0.61–1.54) and allele 4R versus 7R (Random model *Z* = 4.81; *P* < 0.00001, *d* = 1.20 95%CI: 0.71–1.69), respectively. These results showed evidence of decreased functionality of the 7R compared with the 2R and the 4R.

**Table 4:** Summary of in vitro studies assessing functional differences among *DRD4* VNTRs 48 bp.

Authors	References	Years	Technique	Functional response	Cells	Agonist
Asghari V et al.	[1]	1994	[^3^H]spiperone binding RIA	Non-specific	COS-7	Dopamine
Asghari V et al.	[2]	1995	[^3^H]spiperone binding RIA	cAMP inhibition	CHO-K1	Dopamine
Sanyal S & Van Tol HH	[3]	1997	[^3^H]spiperone binding RIA	cAMP inhibition	GH4C1	Dopamine
Oldenhof J et al.	[4]	1998	[^3^H]spiperone binding RIA	cAMP inhibition	CHO-K1	Dopamine
Jovanovic V et al.	[5]	1999	[^3^H]spiperone binding RIA	cAMP inhibition	CHO-K1	Dopamine
Watts VJ et al.	[6]	1999	[^3^H]spiperone binding RIA	cAMP inhibition	HEK 293	Dopamine
Kazmi MA et al.	[7]	2000	[^3^H]spiperone Ca^2+^ channel flux assay	Ca^2+^ channel current inhibition	HEK 293T	Quinpirole
Gilliland SL et al.	[8]	2000	[^35^S]GTPγS agonist stimulated binding assay	G_i_ protein	CHO-K1	Quinpirole
Czermak C et al.	[9]	2006	[^35^S]GTPγS agonist stimulated binding assay	G_i_ protein	CHO-K1	Dopamine
Van Craenenbroeck K et al.	[10]	2011	[^35^S]GTPγS agonist stimulated binding assay	Non-specific	HEK 293T	Quinpirole
Borroto-Escuela DO et al.	[11]	2011	BRET_50_ assay	Receptors ratio	HEK 293T	nr
Van Craenenbroeck K et al.	[10]	2011	BRET_50_ assay	Non-specific	HEK 293T	Quinpirole
Sanchez-Soto M et al.	[12]	2016	BRET_50_ assay	cAMP inhibition	HEK 293T	Dopamine
Sanchez-Soto M et al.	[13]	2018	BRET_50_ assay	G_i_ protein	HEK 293T	Dopamine
Sanyal S & Van Tol HH	[3]	1997	Luciferase reporter assay	cAMP inhibition	GH4C1	Quinpirole
Schoots O & Van Tol HH	[14]	2003	Luciferase reporter assay	Expression	GH4C1	nr
Van Craenenbroeck K et al.	[15]	2005	Western analysis	Expression	CHO-K1	Quinpirole
Gonzalez S et al.	[16]	2012	Transient transfection	MAPK activation (ERK 1/2 phosphorylation)	HEK 293T	RO-10-5824

*RIA* radioimmunoassay, *BRET* bioluminescence resonance energy transfer; *nr* non-reported.

### Bioinformatics in silico analysis

Using the 1000 Genomes Database, we built *DRD4* gene LD blocks for different populations (African, American, East Asian, European and South Asian). We found that the 48 bp VNTR was not tagged by any of the GWAS SNPs used by Demontis and colleagues^15^ (Supplementary Fig. S9).

According to the brain tissues filter, the analysis showed a nominally significant association (*P* < 0.05) with *DRD4* due to a downregulation of gene expression in a specific brain area, which is the Putamen region included in Basal Ganglia (*Z*-score = −3.02, *P* = 0.00252).

## Discussion

### Short summary of the major findings

*DRD4* 48 bp VNTR appears to modulate the ADHD phenotype and MPH response across the lifespan, with differential associations depending on age and populations. This polymorphism has a significant impact on the pathophysiology, much more significant than the common SNPs variants.

### Findings in relation to the literature

In our prior review^32^, we showed that the 7R allele, in childhood, has been associated with specific neuropsychological/neurophysiological tasks, brain structure and altered expression levels of *DRD4*. We also found that the 7R allele seems to moderate the effects of maternal smoking during pregnancy, season of birth, and parenting on externalizing behaviour in ADHD. The present study provides further evidence, with more updated meta-analyses, for the 7R/“long” allele as a strong ADHD susceptibility risk factor in European-Caucasian populations and that this allele leads to reduced biological functionality compared with the 2R and 4R alleles, modulating the receptor’s signal transduction properties and altering intracellular cAMP level^31^. In other words, 7R allele has a reduced potency for coupling dopamine receptors to adenylate cyclase^31^, and consequently a decreased dopamine sensitivity. More importantly, a further recent evidence^55^ explores whether candidate genes are associated with multiple disorders via pleiotropic mechanisms, and/or if other genes are specific to susceptibility for individual psychiatric disorders. Using a meta-analytic approach, the authors found that the 7R allele of *DRD4* was specifically implicated in ADHD and no with any other psychiatric diseases, validating our data both as regards the 7R allele as a major risk susceptibility factor for ADHD and as regards its specificity for ADHD. Of note, it results also specifically associated to childhood ADHD, and not in adult ADHD^7,44^. On the other hand, the 4R/“short” allele was a protective population-specific (European-Caucasian and South American) factor in children with ADHD, whereas our previous data^44^ supported no association in ADHD adulthood in general population.

As associations were observed also for the *SLC6A3* gene^7,55^ where allelic variants showed differential effects in children and adults with ADHD, these findings suggest that *DRD4* and *SLC6A3* are among those genes that account for developmental variations with differential effects across the lifespan.

From the last SNPs/GWAS meta-analysis^15^, five SNPs in *DRD4* were not significant according the GWAS cut-off significance (10^−8^). In this work, we show that those findings do not contradict our conclusions on the role of *DRD4* in ADHD, because none of the SNPs assayed in that study^15^ are in LD with the 48 bp VNTR. Thus, the role played by the *DRD4* in ADHD susceptibility is determined predominantly by the 48 bp VNTR variants.

The population-specific allelic heterogeneity we found is consistent with prior reports that the *DRD4* VNTR displays a high degree of variability across populations worldwide, e.g. 48% in native Americans, but only 0–2% in Asians. There is no commonly accepted explanation for this variability at the *DRD4* locus. A recent review^56^ suggested that the common and probably ancestral allele has four repeats, originating 300,000 years ago, whereas the 7R allele is up to 10 times younger. The 7R allele may have arisen as a rare mutational event and then become a high frequency allele by positive selection at a time of the major expansion of human population (the upper Paleolithic). In this way, individuals with novelty-seeking personality traits may have driven the expansion of the 7R variant, or it may have conferred a reproductive advantage in male-competitive societies. In the Americas, an increase in the 7R allele may have been due to a successive founder effect, and in China a decrease in the 7R may have been due to selective reproduction of males without the 7R allele. At the same time, there appears to be selective forces working to balance the alleles in modern societies (balancing selection), and the prevalence of the 7R allele may now be at a stable level or near a fixation point^56^.

Polymorphisms within key monoaminergic genes have been associated with the response to stimulant medication, albeit through conflicting evidence. This is mechanistically intuitive as MPH modulates extracellular catecholamine levels through interaction with dopaminergic, adrenergic and serotonergic system components. MPH inhibits catecholamine reuptake and modulates dopamine and norepinephrine levels, by binding to and blocking dopamine and norepinephrine transporters, thereby increasing extracellular concentrations^57^.

The most recent pharmacogenetics meta-analysis on the *DRD4* 48 bp VNTR^38^ reported a significant association between MPH efficacy and the 4R allele. ADHD children with 4R/4R genotypes showed a 66% increased chance for efficacious MPH response; compared with others, where the efficacy measure was defined by changes at Clinical Global Impression-Improvement (CGI-I) and Severity (CGI-S), and ADHD Rating Scale (ADHD-RS), whereas the 7R allele versus others did not reach significant association, even though a trend towards to poor MPH response was observed^38^. Thus, these data are in line with the European susceptibility/protection role of 7R“long”/4R alleles, respectively. This is also consistent with the evidence that, as already evidenced, the 4R leads to higher receptor expression and increased sensitivity to dopamine, as compared with the 7R variant. MPH works by blocking the pre-synaptic dopamine transporter, thus increasing synaptic dopamine^58^. Since 7R shows weaker transduction effects, the response to an increased level of synaptic dopamine will be weak^31^. These results further implicate that the children with ADHD homozygotes for 4R alleles would require lower doses of MPH to achieve symptom improvement.

The identification of predictors of pharmacotherapy is needed and always in development, to further the clinical implementation of precision medicine. Of note, patients receiving precision treatment were found to be more medication adherent^59^. Only half of children with ADHD followed pharmacological treatment regimens consistently over the course of a 5-year prospective study, and many reported adverse effects, and also the perceived tolerability may also be an impediment to adherence to treatments. Myer and colleagues^38^ analysed DNA variants in different genes linked to the effectiveness of MPH treatment. Leveraging individual genetic variants within not only *DRD4* but also in *SLC6A2*, *COMT*, *ADRA2A* and *SLC6A3* the authors presented a plausible multivariate to assess risk for poor MPH efficacy. It is possible that, as they suggest, a multivariate predictor would be sufficiently accurate for clinical use. Furthermore, collectively evaluating genetic variability among plausible biological markers for treatment success would eliminate trial-and-error treatment used today^60^.

### Limitations

We found, in some cases, heterogeneity in effect size across studies, and a significant Egger’s test for funnel plot asymmetry which indicates presence of publication bias. Differences in sample and methodological approaches, absence of quality control analyses other than tests of Hardy-Weinberg equilibrium, absence of quality of the genotyping conducted, no repeated genotyping consistency, no call rates, and studies conducted in a wide time lapse (1996–2018), are some reasons for the presence of heterogeneity. Moreover, even though we conducted the analyses taking into consideration different populations^37^, some studies are not based on pure populations: i.e., refs. ^61–74^ are primarily European-Caucasian (about 80%), but the remaining percentage of the sample also contain other ethnic groups (Table 1). Furthermore, even the studies^75–81^ performed in South American populations contains for about 70% Caucasian samples, the remaining percentage is related to African or Native American admixture, Amerindian or Paisa Antioquia community genetic isolate (Table 1).

Other important sources of heterogeneity are linked to how the genotypic classification of alleles was conducted in different studies. Some used 7R carriers vs. non-carriers, others: (2–5) vs. (6–11) repeat carriers; (2–6) vs. (7–11) repeat carriers; (22, 24, 44) vs. (27, 47, 77) genotypes; 2–4 vs 5–11R carriers (for a review, see Pappa and colleagues^45^). We defined “short” allele (to 2R from 4R), and “long” allele (to 5R from 8R), a choice also confirmed by our data because the results did not change, as compared with the 4R and 7R analyses, respectively.

Finally, a TDT study design results significantly less heterogeneous than a CC study. Thus, we suggest conducting the meta-analyses, taking in consideration study design (differently from the previous meta-analyses^35,36^).

Regarding the results from Egger’s test, for the 7R case, we observed presence of publication bias in European populations with a CC model (*P* = 0.018), but the *P* value becomes smaller (*P* = 0.0004) when CC model is merged with TDT study design. We observed that, when we eliminated from the analyses Sonuga-Barke and colleagues^47^ along with Altink and colleagues^48^, the values are less significant and the *P* value for the total sample was 0.83. This could further mean the importance of studying this kind of polymorphism in samples where there are not mixed populations.

### Conclusions and future directions

Our data strongly suggest that *DRD4* 48 bp VNTR could influence the ADHD susceptibility as well as the MPH response across the lifespan, with differential associations depending on age and populations. Interestingly, as compared with the other common SNPs variants, this VNTR polymorphism shows a significant impact on the pathophysiology of ADHD.

The advent of the new and high-throughput technologies such as next generation sequencing are contributing to better elucidate the implication of the rare variants on the ADHD susceptibility: interestingly it has been observed an increased burden of rare variants inside the 7R allele of *DRD4* both in ADHD children^72^, and in adults^75^ that needed further investigation.

In the era of precision medicine, the identification of biomarkers associated to diagnosis and treatment represents a valid way to classify complex mental disorders such as ADHD and offers the opportunity to standardize and improve diagnostic assessment, provide insights into etiological mechanisms, and contribute to developing individualized therapies. Although biomarkers are successfully used in predicting diseases such as cancer, there is no lab test that is used clinically for the diagnosis of ADHD. While there are several pharmacological treatments for ADHD, the mechanisms of action of these agents are still unclear and no specific biological predictors of treatment response are available. We here want to strength the added value provided by the biomarker identification approach for ADHD, and even though future work is needed, we speculate that 7R and 4R alleles of the 48 bp VNTR can contribute to improve the diagnostic picture with their specificity to childhood ADHD and to be a further actor in that possible multivariate predictor^38^ to the MPH response that could be sufficiently accurate for clinical use.

## Supplementary information

Supplementary Fig. S1

Supplementary Fig. S2

Supplementary Fig. S3

Supplementary Fig. S4

Supplementary Fig. S5

Supplementary Fig. S6

Supplementary Fig. S7

Supplementary Fig. S8

Supplementary Fig. S9

Supplementary References for Table 4

Supplementary References Table 1

Supplementary Table S1

Supplementary Table S2

Supplementary material Legends
