# Supplementary material for: DRD4 48 bp multiallelic variants as age-population-specific biomarkers in attention-deficit/hyperactivity disorder
Source: Transl Psychiatry. 2020 Feb 19;10:70. doi: 10.1038/s41398-020-0755-4 (PMC7031506; doi:10.1038/s41398-020-0755-4)
Supplement: Supplementary file 1 — Supplementary Fig. S1 [file 41398_2020_755_MOESM1_ESM.pptx]

## Slide 1
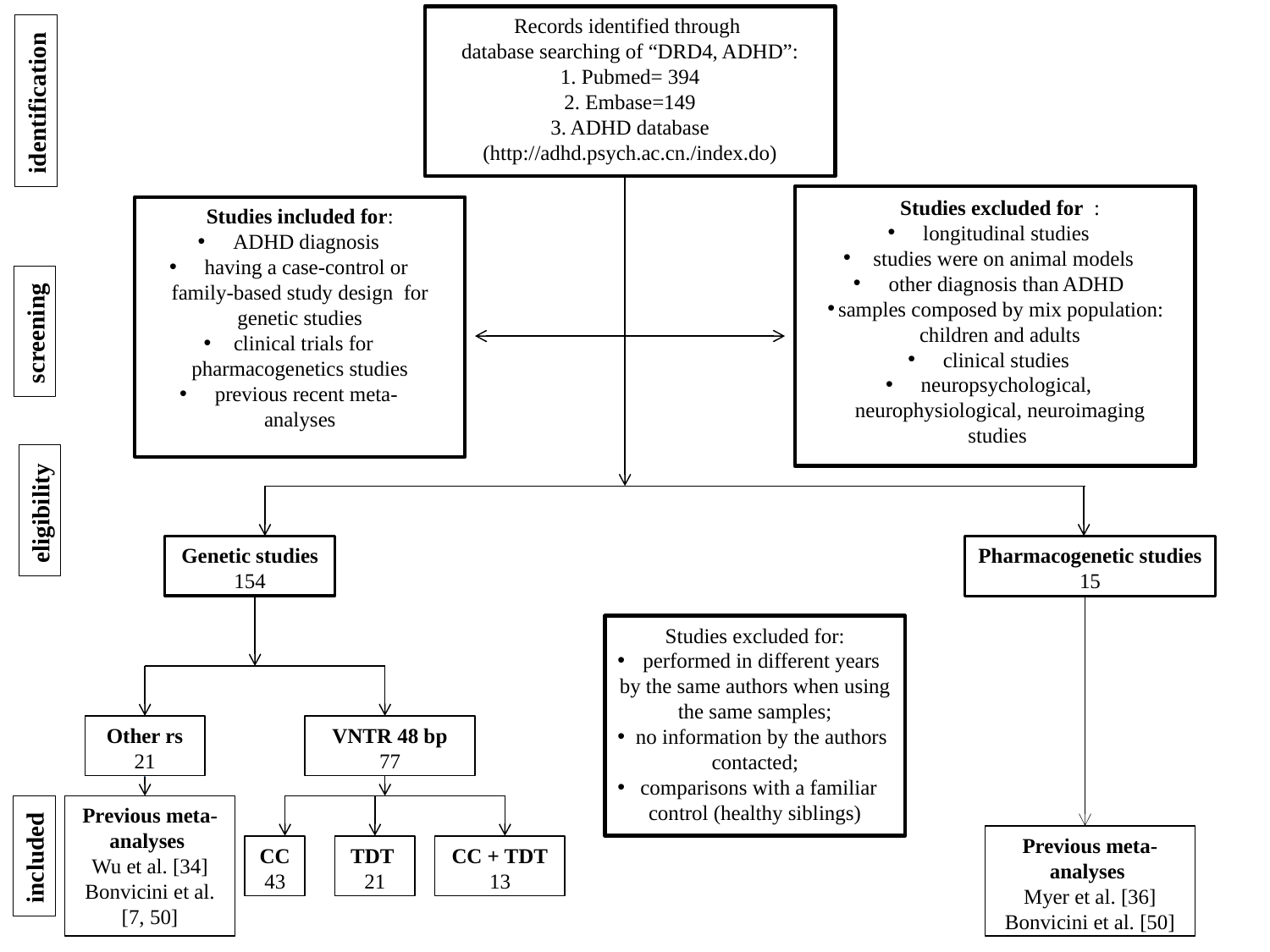

Records identified through
database searching of “DRD4, ADHD”:
1. Pubmed= 394
2. Embase=149
3. ADHD database (http://adhd.psych.ac.cn./index.do)
identification
Studies excluded for :
 longitudinal studies
studies were on animal models
 other diagnosis than ADHD
samples composed by mix population:
children and adults
 clinical studies
 neuropsychological, neurophysiological, neuroimaging studies
Studies included for:
 ADHD diagnosis
 having a case-control or family-based study design for genetic studies
clinical trials for pharmacogenetics studies
 previous recent meta-analyses
screening
eligibility
Genetic studies
154
Pharmacogenetic studies
15
Studies excluded for:
 performed in different years by the same authors when using the same samples;
 no information by the authors contacted;
comparisons with a familiar control (healthy siblings)
Other rs
21
VNTR 48 bp
77
Previous meta-analyses
Wu et al. [34]
Bonvicini et al. [7, 50]
Previous meta-analyses
Myer et al. [36]
Bonvicini et al. [50]
included
TDT
21
CC 43
CC + TDT
13
