# Supplementary figures and images for: DRD4 48 bp multiallelic variants as age-population-specific biomarkers in attention-deficit/hyperactivity disorder
Source: Transl Psychiatry. 2020 Feb 19;10:70. doi: 10.1038/s41398-020-0755-4 (PMC7031506; doi:10.1038/s41398-020-0755-4)

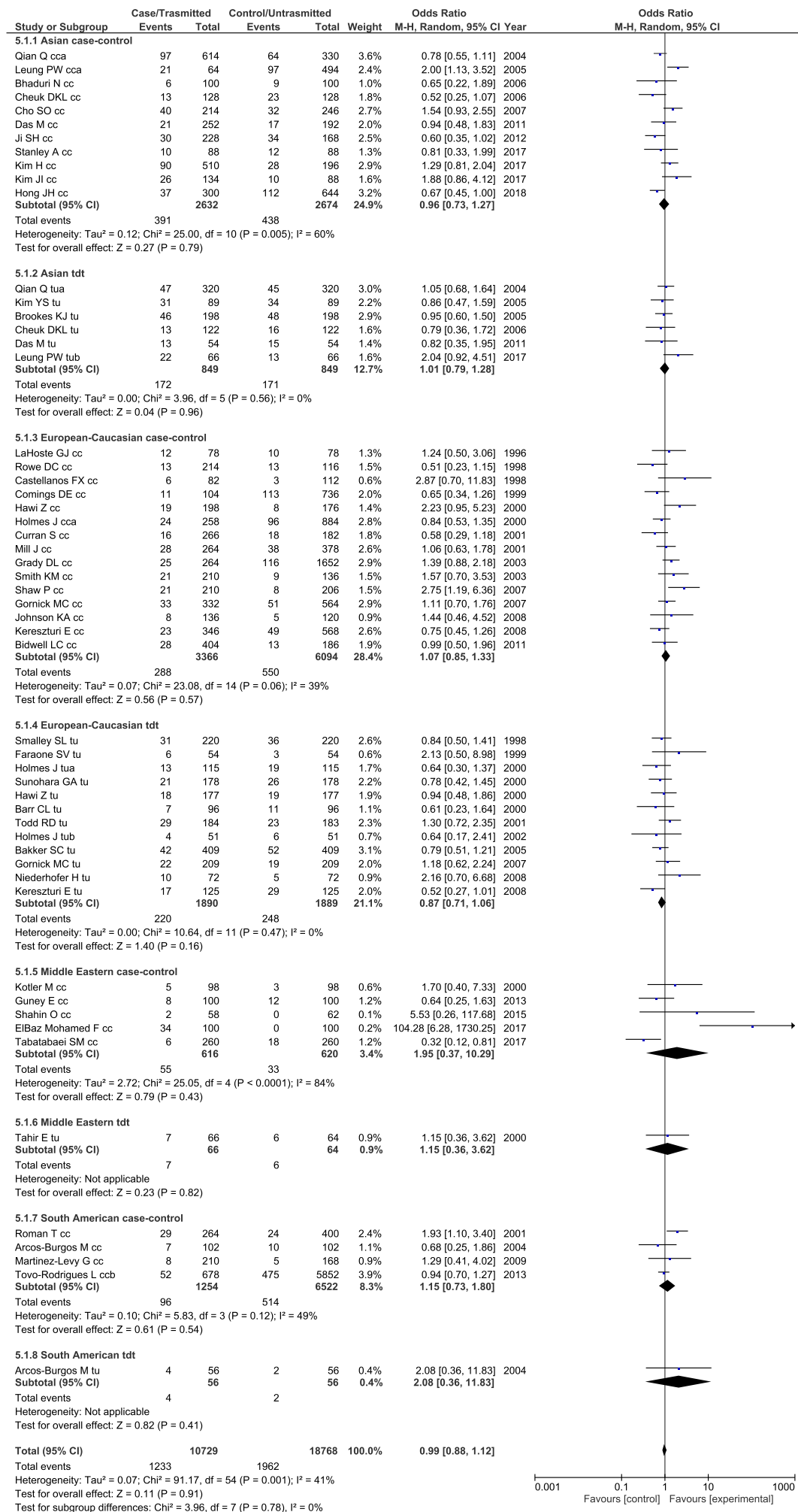

Supplement: Supplementary file 2 — Supplementary Fig. S2 [file 41398_2020_755_MOESM2_ESM.pdf]

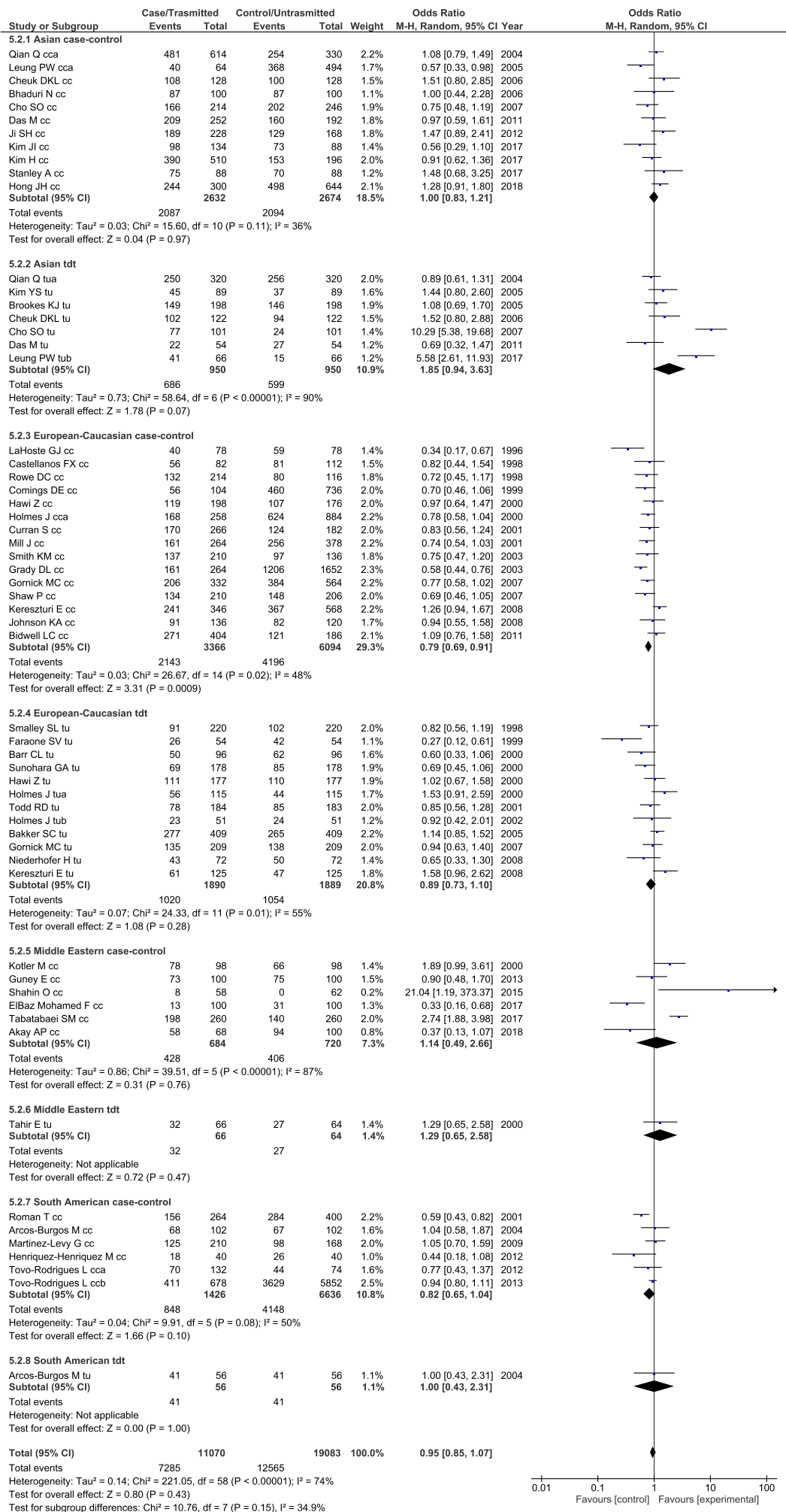

Supplement: Supplementary file 3 — Supplementary Fig. S3 [file 41398_2020_755_MOESM3_ESM.pdf]

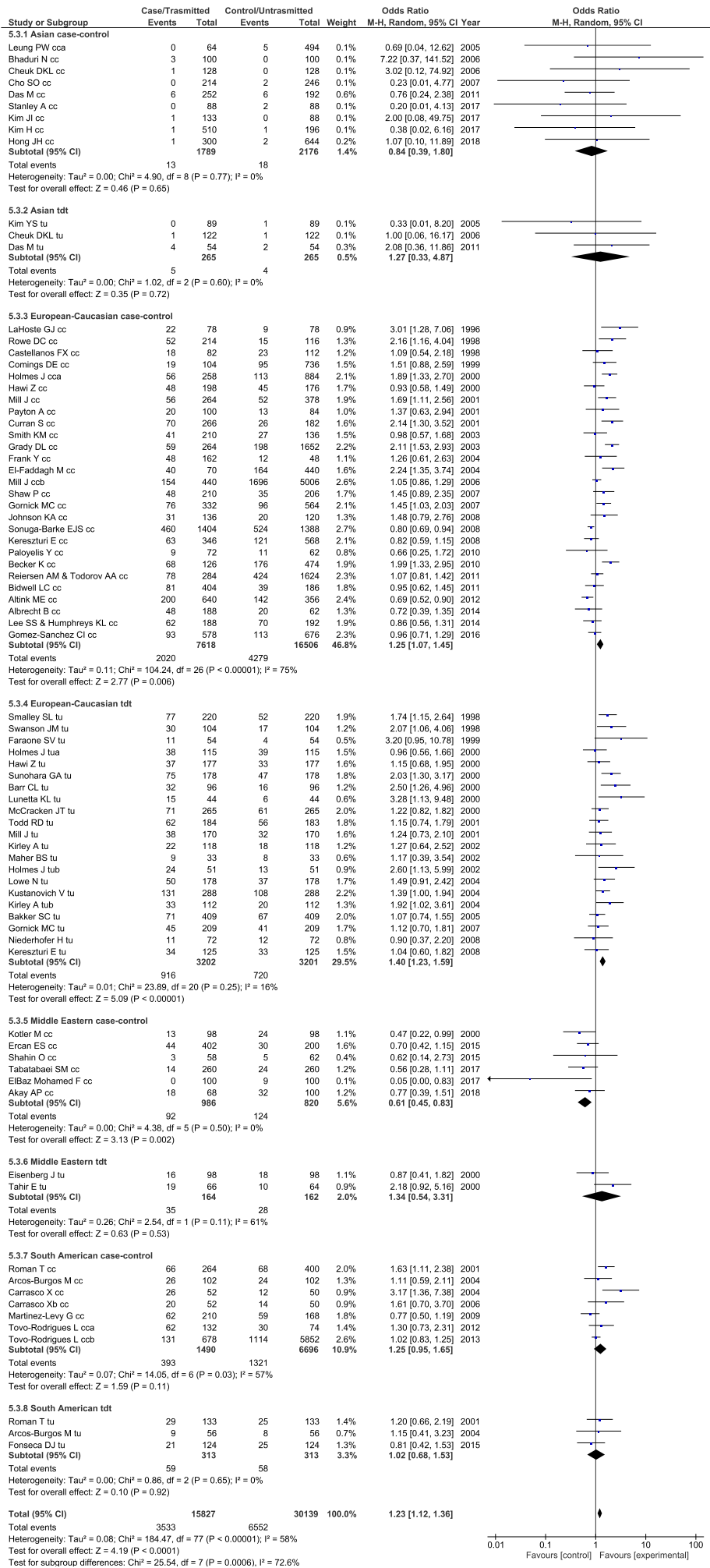

Supplement: Supplementary file 4 — Supplementary Fig. S4 [file 41398_2020_755_MOESM4_ESM.pdf]

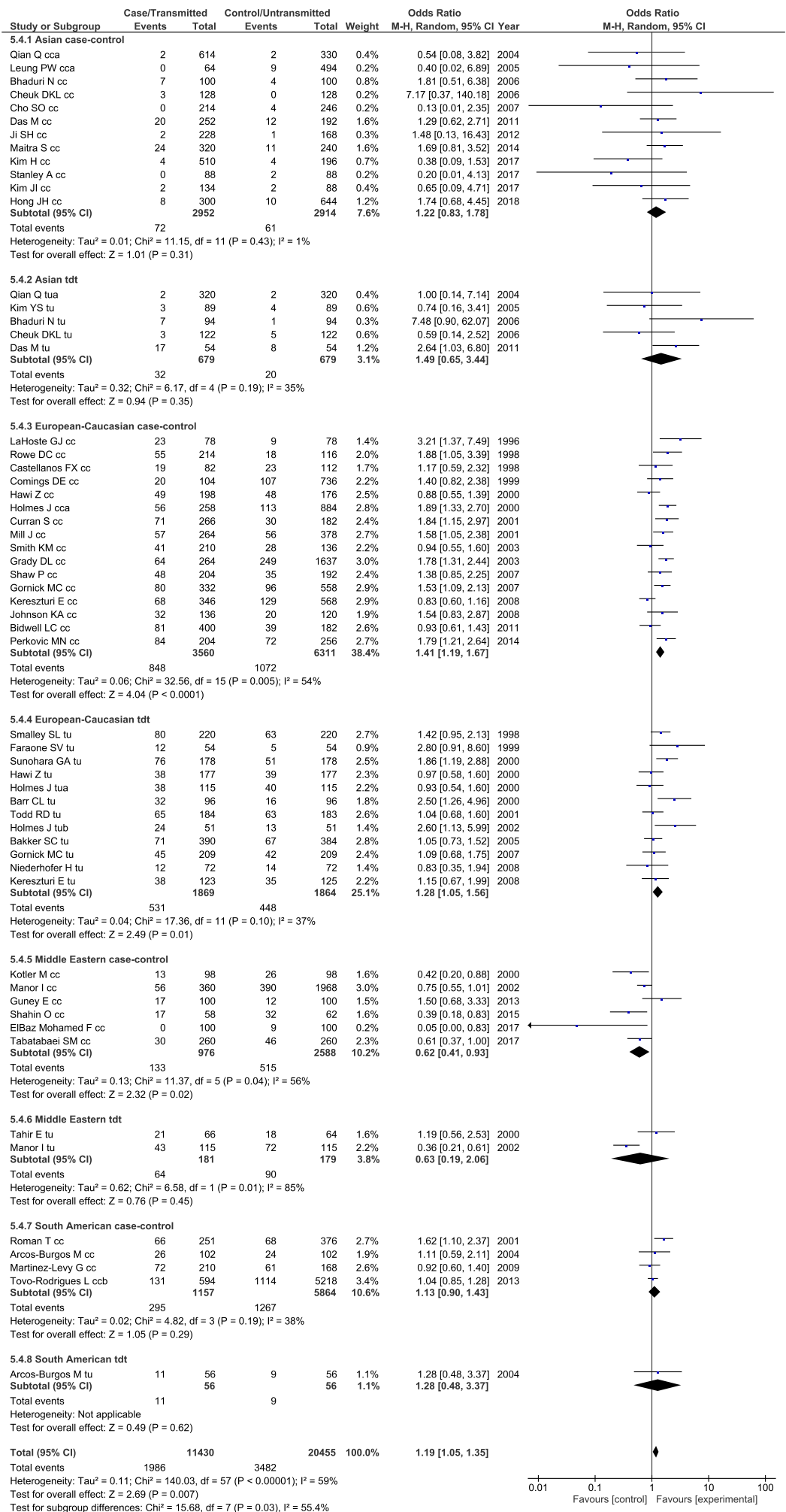

Supplement: Supplementary file 5 — Supplementary Fig. S5 [file 41398_2020_755_MOESM5_ESM.pdf]

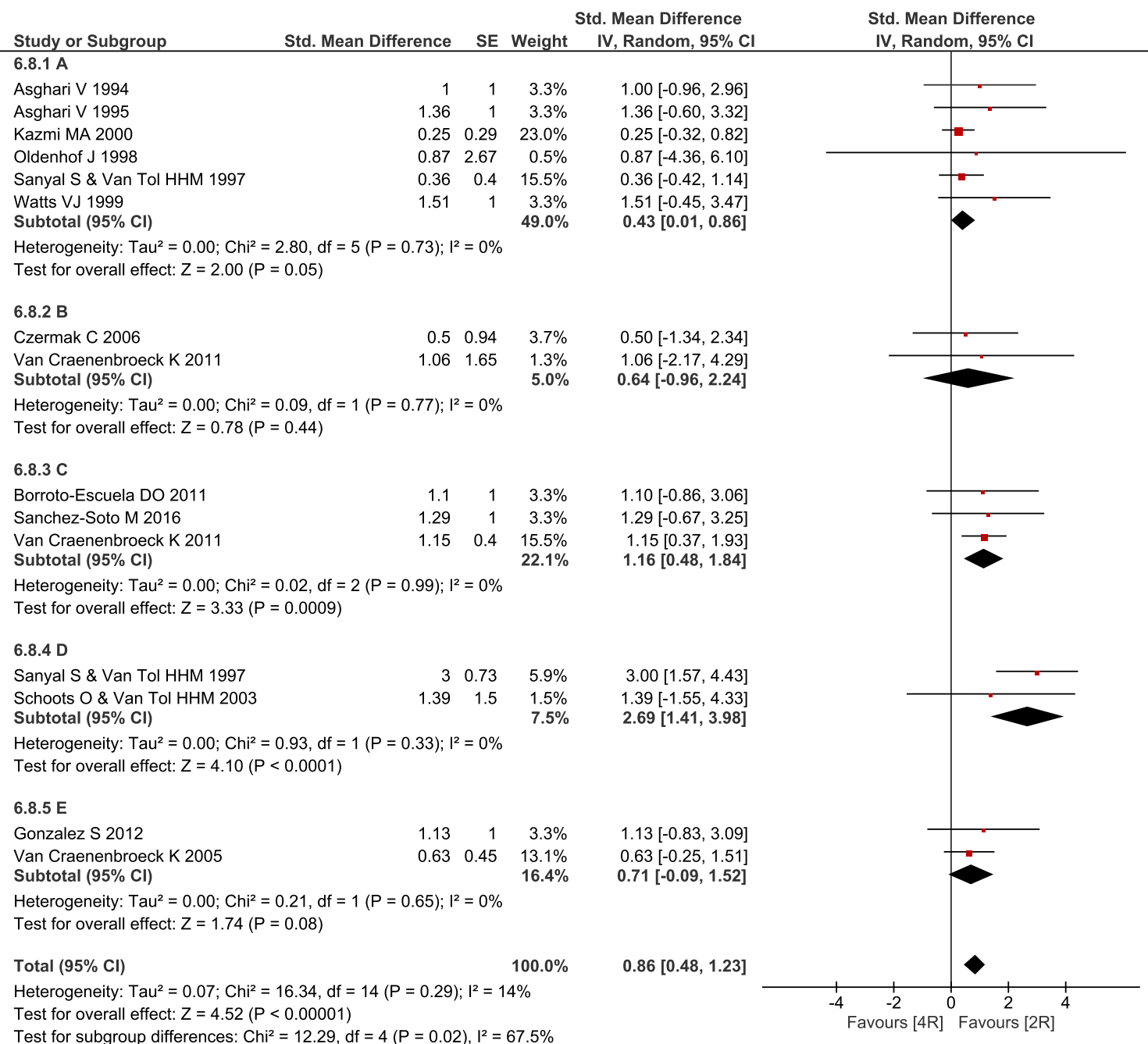

Supplement: Supplementary file 6 — Supplementary Fig. S6 [file 41398_2020_755_MOESM6_ESM.pdf]

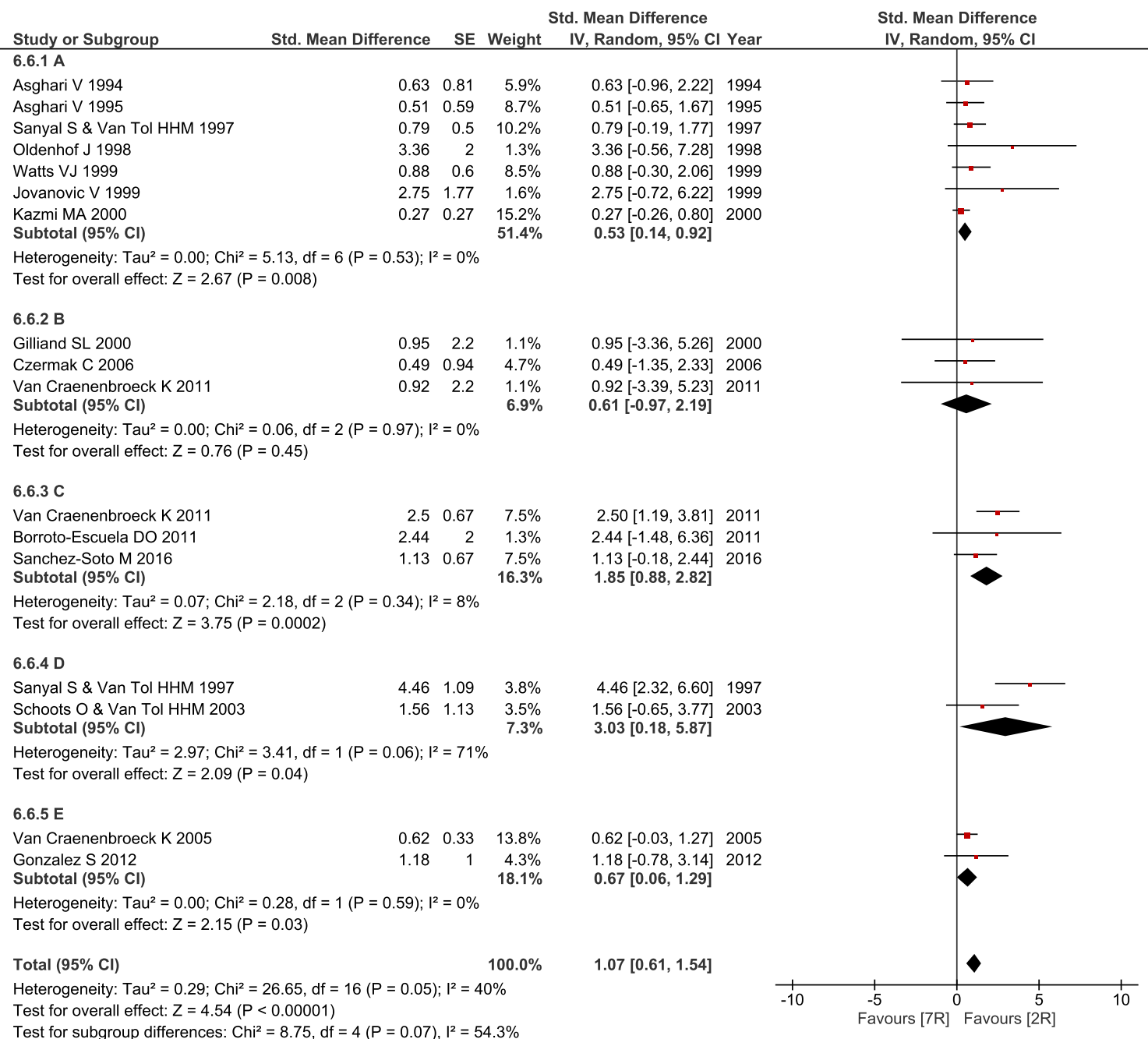

Supplement: Supplementary file 7 — Supplementary Fig. S7 [file 41398_2020_755_MOESM7_ESM.pdf]

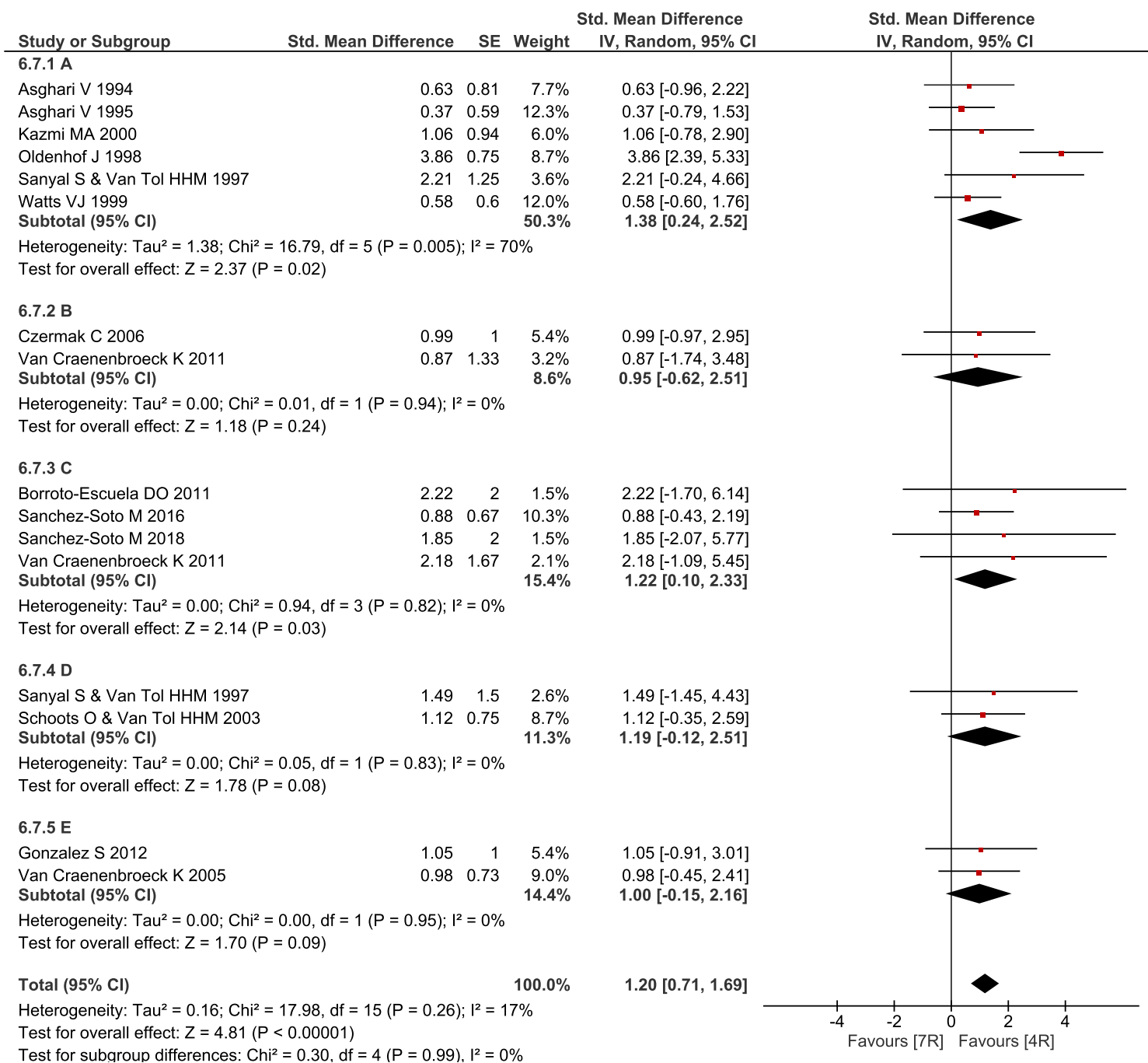

Supplement: Supplementary file 8 — Supplementary Fig. S8 [file 41398_2020_755_MOESM8_ESM.pdf]

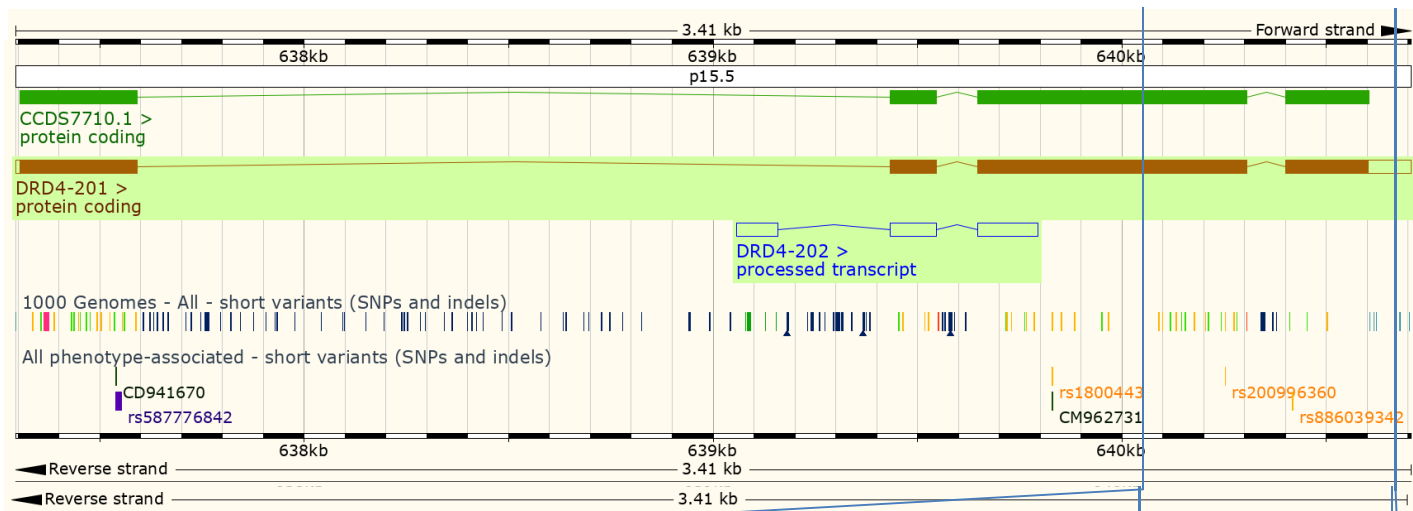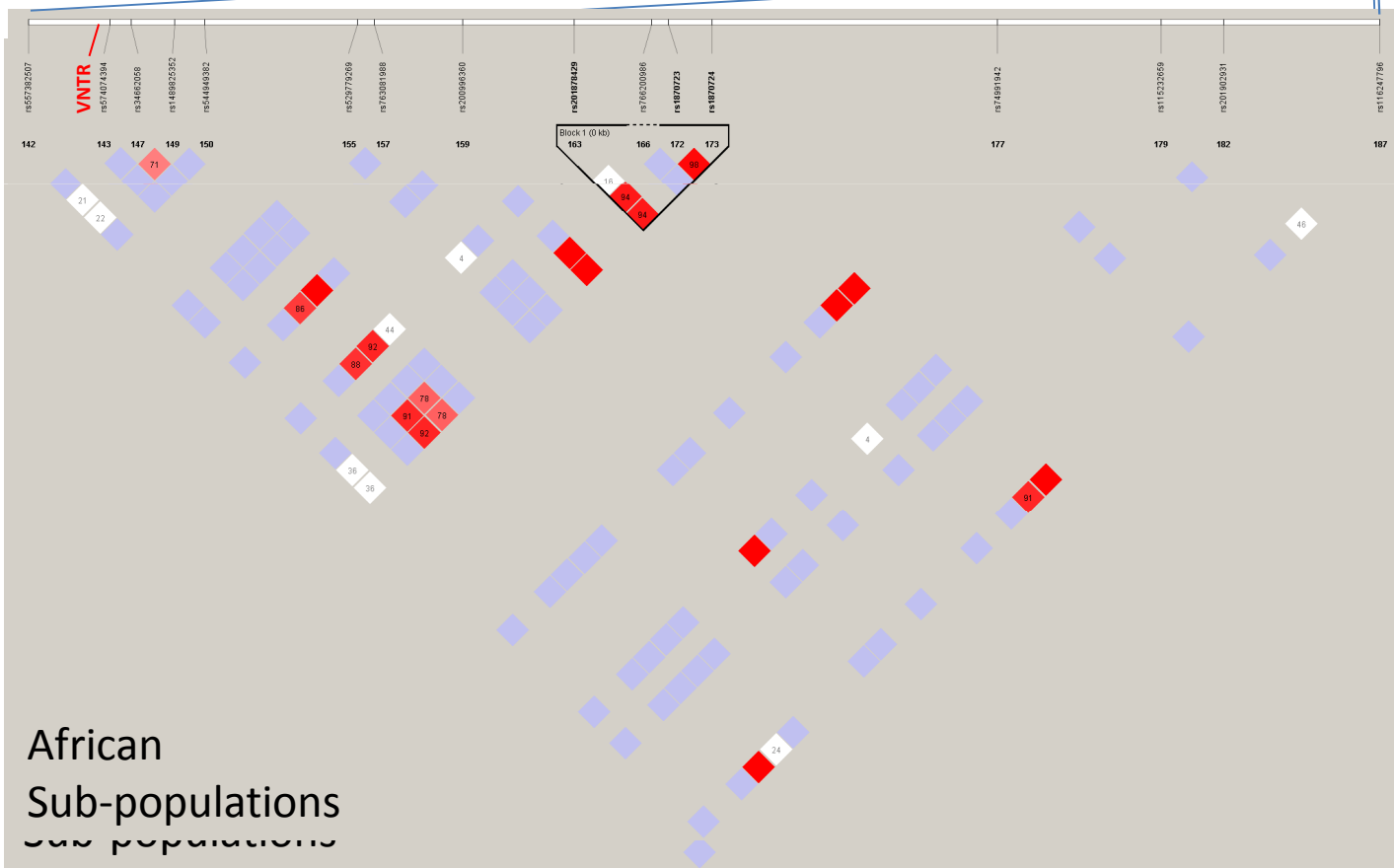

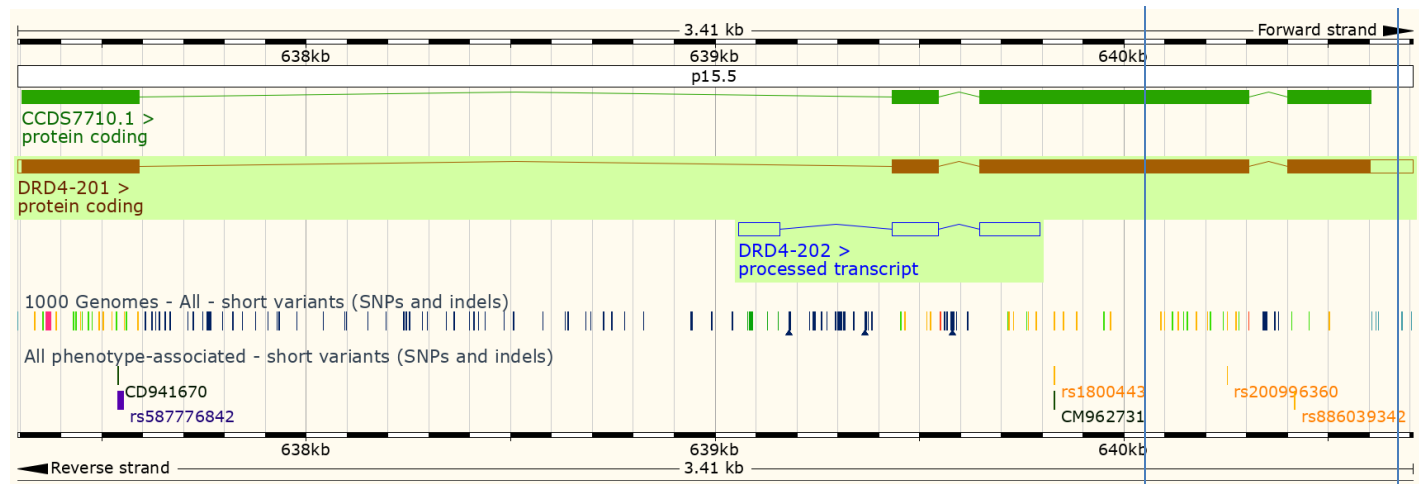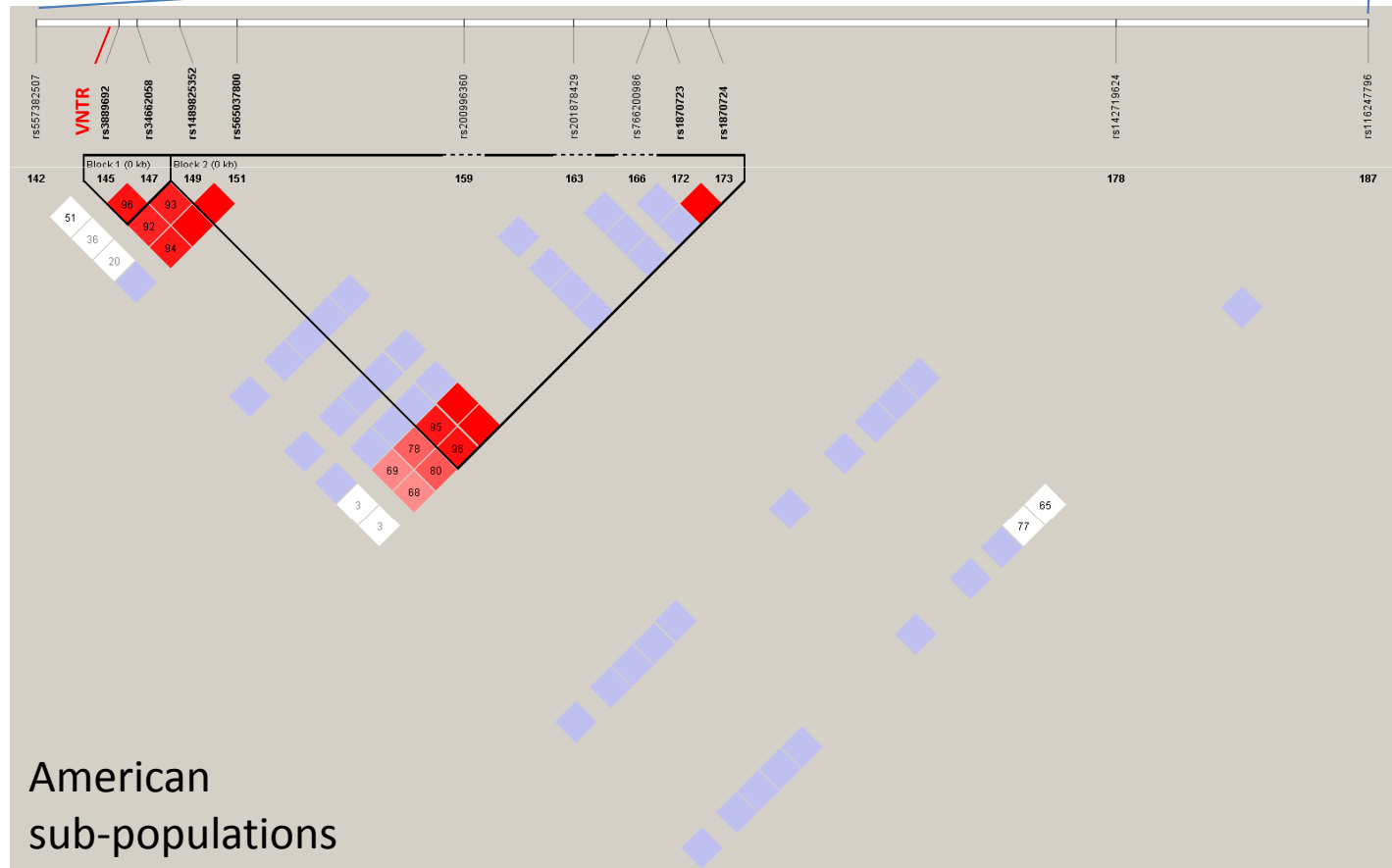

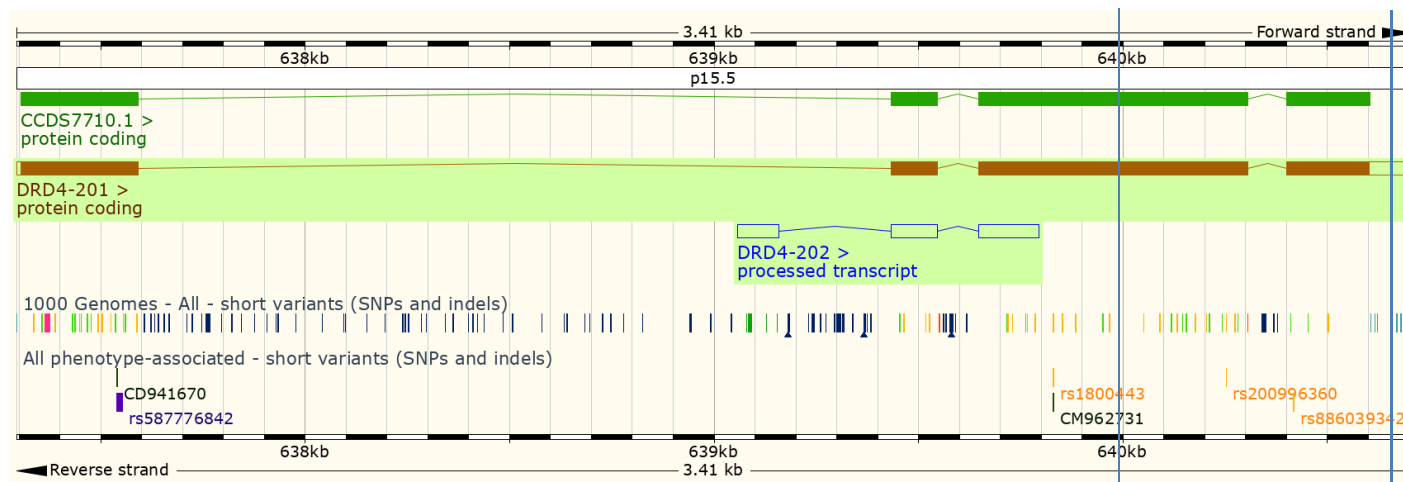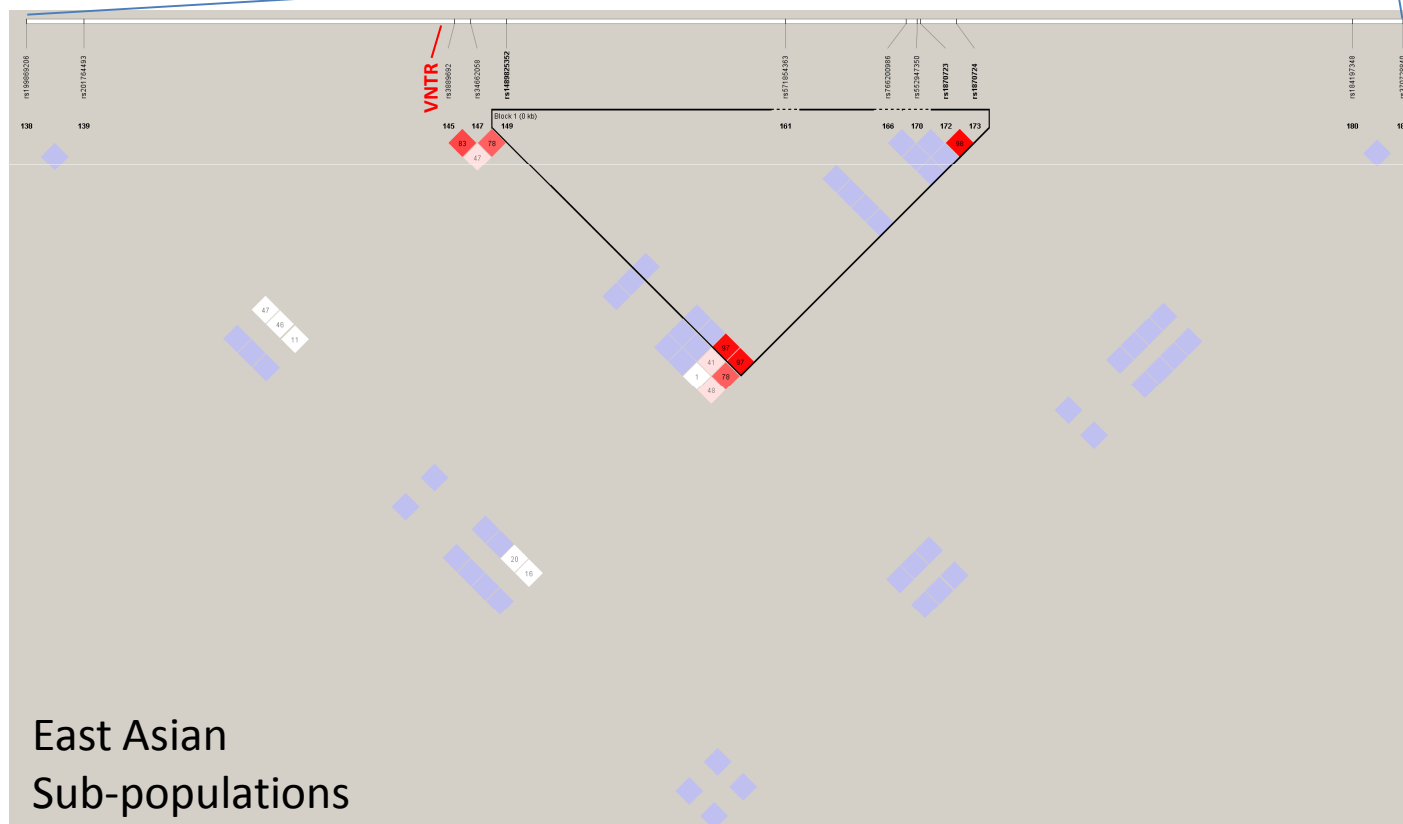

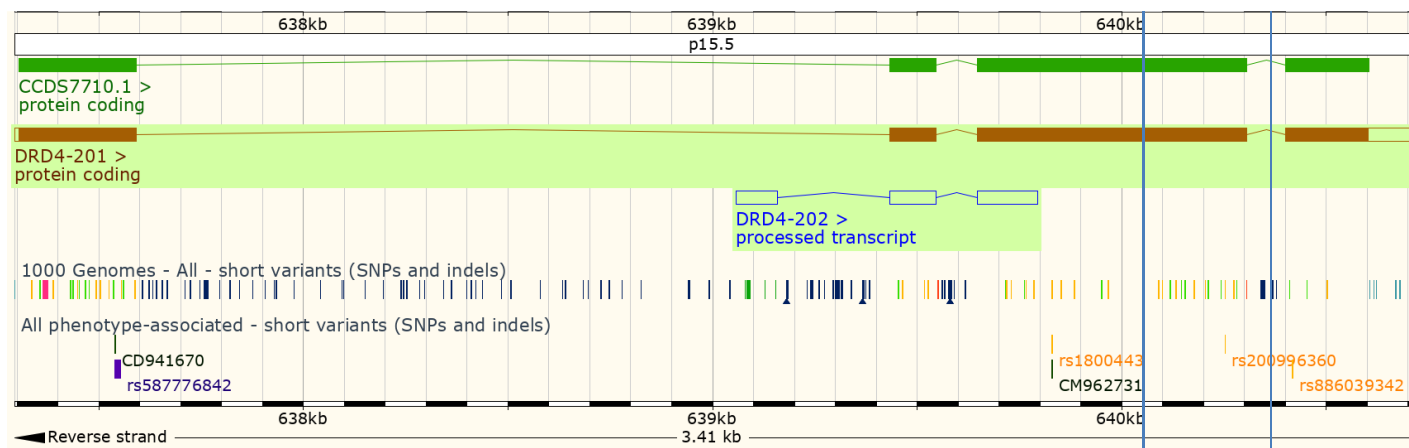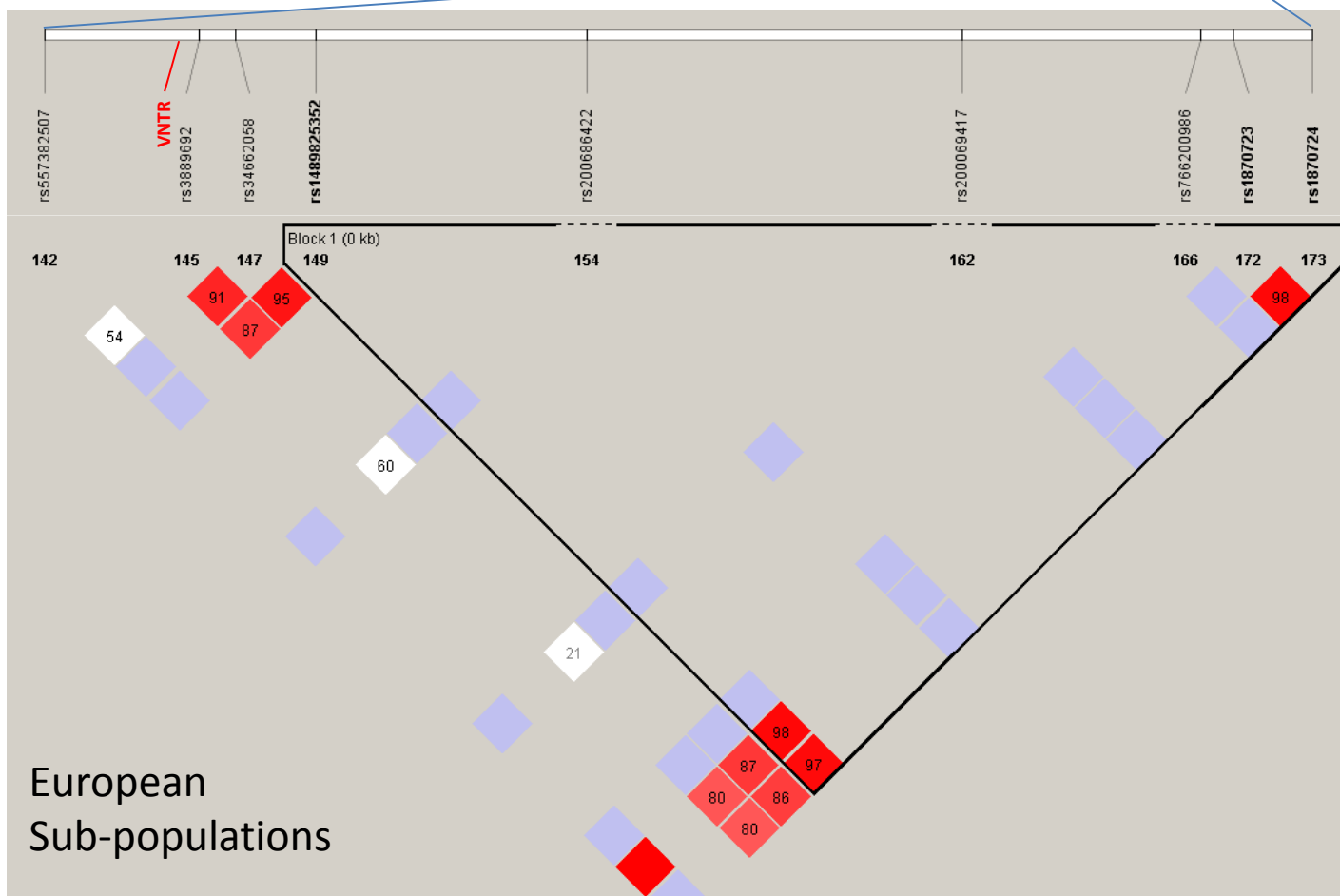

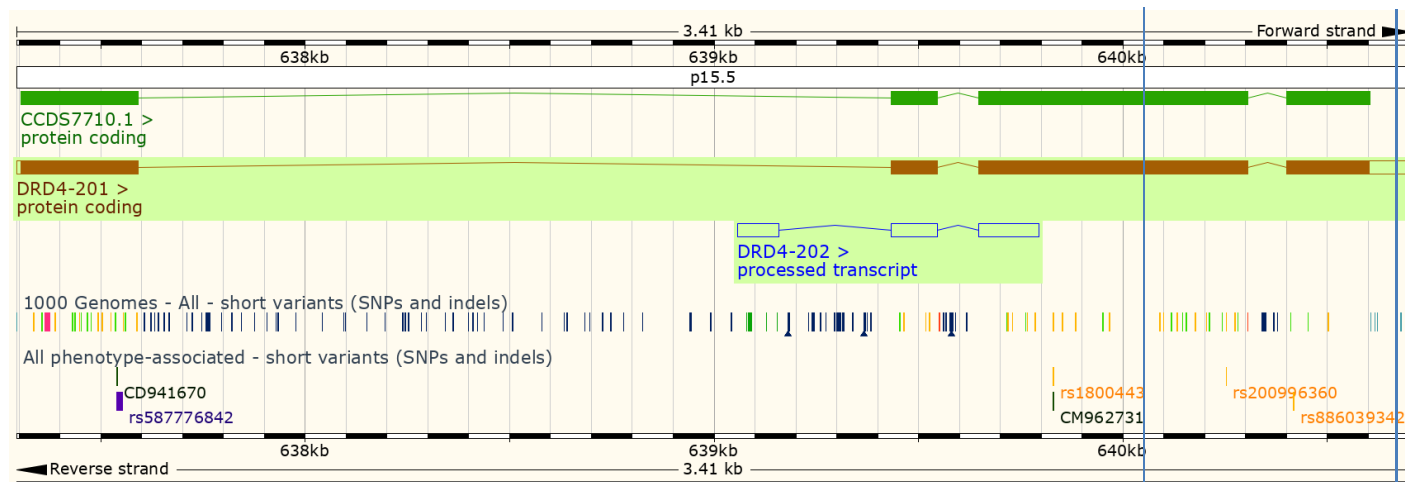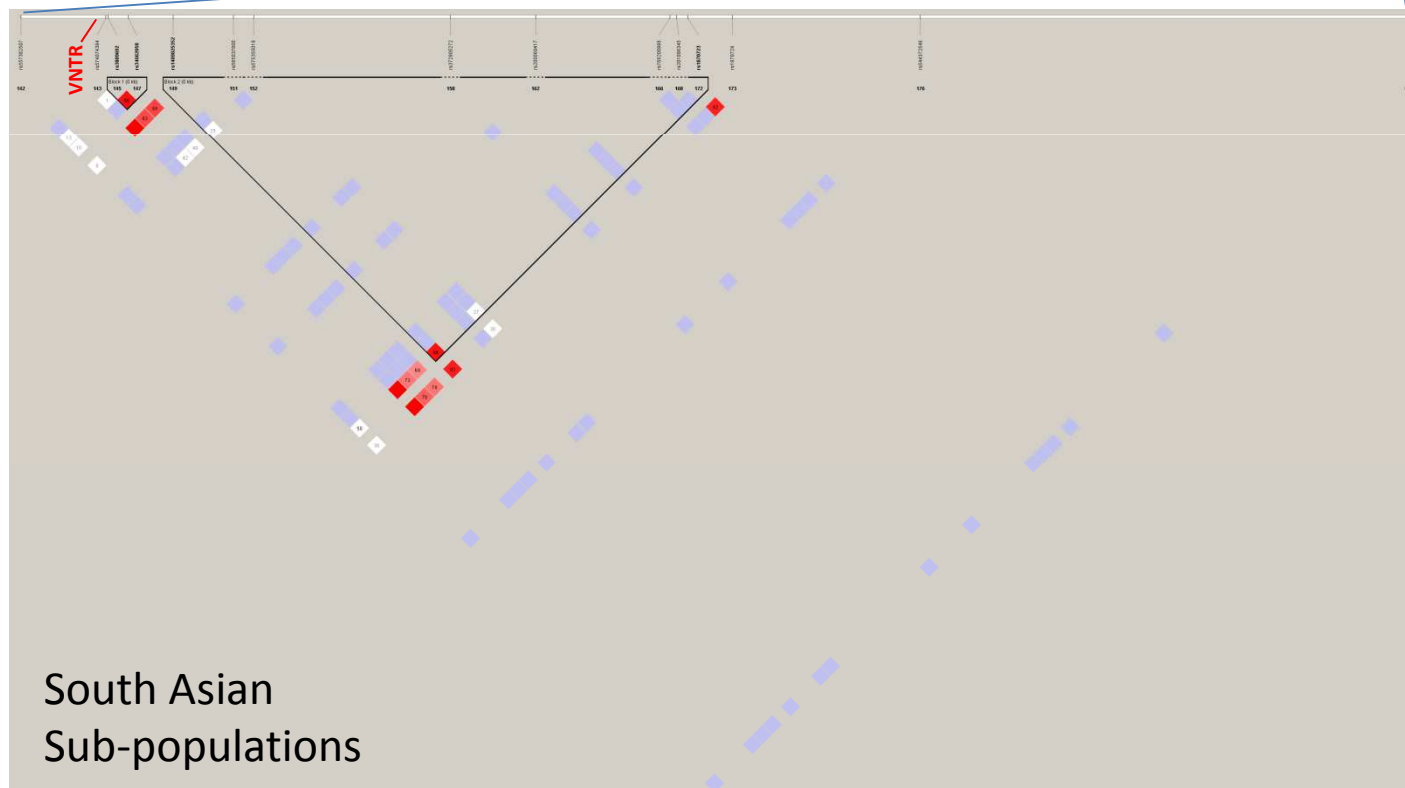

Supplement: Supplementary file 9 — Supplementary Fig. S9 [file 41398_2020_755_MOESM9_ESM.pdf]
