# Supplementary material for: DRD4 48 bp multiallelic variants as age-population-specific biomarkers in attention-deficit/hyperactivity disorder
Source: Transl Psychiatry. 2020 Feb 19;10:70. doi: 10.1038/s41398-020-0755-4 (PMC7031506; doi:10.1038/s41398-020-0755-4)
Supplement: Supplementary file 10 — Supplementary References for Table 4 [file 41398_2020_755_MOESM10_ESM.docx]

**References**

1. Asghari V, Schoots O, van Kats S, Ohara K, Jovanovic V, Guan HC, et al. Dopamine D4 receptor repeat: Analysis of different native and mutant forms of the human and rat genes. Mol Pharmacol 1994; **46:** 364-73.

2. Asghari V, Sanyal S, Buchwaldt S, Paterson A, Jovanovic V, Van Tol HH. Modulation of intracellular cyclic AMP levels by different human dopamine D4 receptor variants. J Neurochem 1995; **65:** 1157-65.

3. Sanyal S, Van Tol HH. Dopamine D4 receptor-mediated inhibition of cyclic adenosine 3',5'-monophosphate production does not affect prolactin regulation. Endocrinology 1997; **138:** 1871-8.

4. Oldenhof J, Vickery R, Anafi M, Oak J, Ray A, Schoots O, et al. SH3 binding domains in the dopamine D4 receptor. Biochemistry 1998; **37:** 15726-36.

5. Jovanovic V, Guan HC, Van Tol HH. Comparative pharmacological and functional analysis of the human dopamine D4.2 and D4.10 receptor variants. Pharmacogenetics 1999; **9:** 561-8.

6. Watts VJ, Vu MN, Wiens BL, Jovanovic V, Van Tol HH, Neve KA. Short- and long-term heterologous sensitization of adenylate cyclase by D4 dopamine receptors. Psychopharmacology (Berl) 1999; **141:** 83-92.

7. Kazmi MA, Snyder LA, Cypess AM, Graber SG, Sakmar TP. Selective reconstitution of human D4 dopamine receptor variants with gi alpha subtypes. Biochemistry 2000; **39:** 3734-44.

8. Gilliland SL, Alper RH. Characterization of dopaminergic compounds at hD2short, hD4.2 and hD4.7 receptors in agonist-stimulated [35S]GTPgammaS binding assays. Naunyn Schmiedebergs Arch Pharmacol 2000; **361:** 498-504.

9. Czermak C, Lehofer M, Liebmann PM, Traynor J. 35S]GTPgammaS binding at the human dopamine D4 receptor variants hD4.2, hD4.4 and hD4.7 following stimulation by dopamine, epinephrine and norepinephrine. Eur J Pharmacol 2006; **531:** 20-4.

10. Van Craenenbroeck K, Borroto-Escuela DO, Romero-Fernandez W, Skieterska K, Rondou P, Lintermans B, et al. Dopamine D4 receptor oligomerization--contribution to receptor biogenesis. FEBS J 2011; **278:** 1333-44.

11. Borroto-Escuela DO, Van Craenenbroeck K, Romero-Fernandez W, Guidolin D, Woods AS, Rivera A, et al. Dopamine D2 and D4 receptor heteromerization and its allosteric receptor-receptor interactions. Biochem Biophys Res Commun 2011; **404:** 928-34.

12. Sanchez-Soto M, Bonifazi A, Cai NS, Ellenberger MP, Newman AH, Ferre S, et al. Evidence for noncanonical neurotransmitter activation: Norepinephrine as a dopamine D2-like receptor agonist. Mol Pharmacol 2016; **89:** 457-66.

13. Sanchez-Soto M, Yano H, Cai NS, Casado-Anguera V, Moreno E, Casado V, et al. Revisiting the functional role of dopamine D4 receptor gene polymorphisms: Heteromerization-dependent gain of function of the D4.7 receptor variant. Mol Neurobiol 2018; .

14. Schoots O, Van Tol HH. The human dopamine D4 receptor repeat sequences modulate expression. Pharmacogenomics J 2003; **3:** 343-8.

15. Van Craenenbroeck K, Clark SD, Cox MJ, Oak JN, Liu F, Van Tol HH. Folding efficiency is rate-limiting in dopamine D4 receptor biogenesis. J Biol Chem 2005; **280:** 19350-7.

16. Gonzalez S, Rangel-Barajas C, Peper M, Lorenzo R, Moreno E, Ciruela F, et al. Dopamine D4 receptor, but not the ADHD-associated D4.7 variant, forms functional heteromers with the dopamine D2S receptor in the brain. Mol Psychiatry 2012; **17:** 650-62.
