# Supplementary material for: DRD4 48 bp multiallelic variants as age-population-specific biomarkers in attention-deficit/hyperactivity disorder
Source: Transl Psychiatry. 2020 Feb 19;10:70. doi: 10.1038/s41398-020-0755-4 (PMC7031506; doi:10.1038/s41398-020-0755-4)
Supplement: Supplementary file 11 — Supplementary References Table 1 [file 41398_2020_755_MOESM11_ESM.docx]

**References**

1. Qian Q, Wang Y, Zhou R, Yang L, Faraone SV. Family-based and case-control association studies of DRD4 and DAT1 polymorphisms in chinese attention deficit hyperactivity disorder patients suggest long repeats contribute to genetic risk for the disorder. Am J Med Genet B Neuropsychiatr Genet 2004; **128B:** 84-9.

2. Leung PW, Lee CC, Hung SF, Ho TP, Tang CP, Kwong SL, et al. Dopamine receptor D4 (DRD4) gene in han chinese children with attention-deficit/hyperactivity disorder (ADHD): Increased prevalence of the 2-repeat allele. Am J Med Genet B Neuropsychiatr Genet 2005; **133B:** 54-6.

3. Cheuk DK, Li SY, Wong V. Exon 3 polymorphisms of dopamine D4 receptor (DRD4) gene and attention deficit hyperactivity disorder in chinese children. Am J Med Genet B Neuropsychiatr Genet 2006; **141B:** 907-11.

4. Leung PW, Chan JK, Chen LH, Lee CC, Hung SF, Ho TP, et al. Family-based association study of DRD4 gene in methylphenidate-responded attention deficit/hyperactivity disorder. PLoS One 2017; **12:** e0173748.

5. Bhaduri N, Das M, Sinha S, Chattopadhyay A, Gangopadhyay PK, Chaudhuri K, et al. Association of dopamine D4 receptor (DRD4) polymorphisms with attention deficit hyperactivity disorder in indian population. Am J Med Genet B Neuropsychiatr Genet 2006; **141B:** 61-6.

6. Das M, Das Bhowmik A, Bhaduri N, Sarkar K, Ghosh P, Sinha S, et al. Role of gene-gene/gene-environment interaction in the etiology of eastern indian ADHD probands. Prog Neuropsychopharmacol Biol Psychiatry 2011; **35:** 577-87.

7. Maitra S, Sarkar K, Ghosh P, Karmakar A, Bhattacharjee A, Sinha S, et al. Potential contribution of dopaminergic gene variants in ADHD core traits and co-morbidity: A study on eastern indian probands. Cell Mol Neurobiol 2014; **34:** 549-64.

8. Stanley A, Chavda K, Subramanian A, Prabhu SV, Ashavaid TF. DRD4 and DAT1 VNTR genotyping in children with attention deficit hyperactivity disorder. Indian J Clin Biochem 2017; **32:** 239-42.

9. Kim YS, Leventhal BL, Kim SJ, Kim BN, Cheon KA, Yoo HJ, et al. Family-based association study of DAT1 and DRD4 polymorphism in korean children with ADHD. Neurosci Lett 2005; **390:** 176-81.

10. Cho SC, Park TW, Kim JW, Yoo HJ, Kim BN, Shin MS, et al. No association of DRD4 exon III polymorphism with attention-deficit hyperactivity disorder in korean children population. Journal of the Korean Academy of Child and Adolescent Psychiatry 2007; **18:** 154-61.

11. Ji HS, Paik KC, Park WS, Lim MH. No association between the response to methylphenidate and DRD4 gene polymorphism in korean attention deficit hyperactivity disorder: A case control study. Clin Psychopharmacol Neurosci 2013; **11:** 13-7.

12. Kim H, Kim JI, Kim H, Kim JW, Kim BN. Interaction effects of GIT1 and DRD4 gene variants on continuous performance test variables in patients with ADHD. Brain Behav 2017; **7:** e00785.

13. Kim JI, Yoo JH, Kim D, Jeong B, Kim BN. The effects of GRIN2B and DRD4 gene variants on local functional connectivity in attention-deficit/hyperactivity disorder. Brain Imaging Behav 2018; **12:** 247-57.

14. Hong JH, Hwang IW, Lim MH, Kwon HJ, Jin HJ. Genetic associations between ADHD and dopaminergic genes (DAT1 and DRD4) VNTRs in korean children. Genes Genomics 2018; **40:** 1309-17.

15. Brookes KJ, Xu X, Chen CK, Huang YS, Wu YY, Asherson P. No evidence for the association of DRD4 with ADHD in a taiwanese population within-family study. BMC Med Genet 2005; **6:** 31,2350-6-31.

16. LaHoste GJ, Swanson JM, Wigal SB, Glabe C, Wigal T, King N, et al. Dopamine D4 receptor gene polymorphism is associated with attention deficit hyperactivity disorder. Mol Psychiatry 1996; **1:** 121-4.

17. Nikolac Perkovic M, Nedic Erjavec G, Stefulj J, Muck-Seler D, Pivac N, Kocijan Hercigonja D, et al. Association between the polymorphisms of the selected genes encoding dopaminergic system with ADHD and autism. Psychiatry Res 2014; **215:** 260-1.

18. Bakker SC, van der Meulen EM, Oteman N, Schelleman H, Pearson PL, Buitelaar JK, et al. DAT1, DRD4, and DRD5 polymorphisms are not associated with ADHD in dutch families. Am J Med Genet B Neuropsychiatr Genet 2005; **132B:** 50-2.

19. Altink ME, Rommelse NN, Slaats-Willemse DI, Vasquez AA, Franke B, Buschgens CJ, et al. The dopamine receptor D4 7-repeat allele influences neurocognitive functioning, but this effect is moderated by age and ADHD status: An exploratory study. World J Biol Psychiatry 2012; **13:** 293-305.

20. El-Faddagh M, Laucht M, Maras A, Vohringer L, Schmidt MH. Association of dopamine D4 receptor (DRD4) gene with attention-deficit/hyperactivity disorder (ADHD) in a high-risk community sample: A longitudinal study from birth to 11 years of age. J Neural Transm (Vienna) 2004; **111:** 883-9.

21. Becker K, Blomeyer D, El-Faddagh M, Esser G, Schmidt MH, Banaschewski T, et al. From regulatory problems in infancy to attention-deficit/hyperactivity disorder in childhood: A moderating role for the dopamine D4 receptor gene? J Pediatr 2010; **156:** 798,803, 803.e1-803.e2.

22. Niederhofer H, Menzel F, Gobel K, Hackenberg B, Richter R, Walter MH, et al. A preliminary report of the dopamine receptor D(4) and the dopamine transporter 1 gene polymorphism and its association with attention deficit hyperactivity disorder. Neuropsychiatr Dis Treat 2008; **4:** 701-5.

23. Albrecht B, Brandeis D, Uebel-von Sandersleben H, Valko L, Heinrich H, Xu X, et al. Genetics of preparation and response control in ADHD: The role of DRD4 and DAT1. J Child Psychol Psychiatry 2014; **55:** 914-23.

24. Kereszturi E, Tarnok Z, Bognar E, Lakatos K, Farkas L, Gadoros J, et al. Catechol-O-methyltransferase Val158Met polymorphism is associated with methylphenidate response in ADHD children. Am J Med Genet B Neuropsychiatr Genet 2008; **147B:** 1431-5.

25. Sonuga-Barke EJ, Brookes KJ, Buitelaar J, Anney R, Bitsakou P, Baeyens D, et al. Intelligence in DSM-IV combined type attention-deficit/hyperactivity disorder is not predicted by either dopamine receptor/transporter genes or other previously identified risk alleles for attention-deficit/hyperactivity disorder. Am J Med Genet B Neuropsychiatr Genet 2008; **147:** 316-9.

26. Hawi Z, McCarron M, Kirley A, Daly G, Fitzgerald M, Gill M. No association of the dopamine DRD4 receptor (DRD4) gene polymorphism with attention deficit hyperactivity disorder (ADHD) in the irish population. Am J Med Genet 2000; **96:** 268-72.

27. Kirley A, Hawi Z, Daly G, McCarron M, Mullins C, Millar N, et al. Dopaminergic system genes in ADHD: Toward a biological hypothesis. Neuropsychopharmacology 2002; **27:** 607-19.

28. Lowe N, Kirley A, Mullins C, Fitzgerald M, Gill M, Hawi Z. Multiple marker analysis at the promoter region of the DRD4 gene and ADHD: Evidence of linkage and association with the SNP -616. Am J Med Genet B Neuropsychiatr Genet 2004; **131B:** 33-7.

29. Johnson KA, Kelly SP, Robertson IH, Barry E, Mulligan A, Daly M, et al. Absence of the 7-repeat variant of the DRD4 VNTR is associated with drifting sustained attention in children with ADHD but not in controls. Am J Med Genet B Neuropsychiatr Genet 2008; **147B:** 927-37.

30. Gomez-Sanchez CI, Riveiro-Alvarez R, Soto-Insuga V, Rodrigo M, Tirado-Requero P, Mahillo-Fernandez I, et al. Attention deficit hyperactivity disorder: Genetic association study in a cohort of spanish children. Behav Brain Funct 2016; **12:** 2,015-0084-6.

31. Holmes J, Payton A, Barrett JH, Hever T, Fitzpatrick H, Trumper AL, et al. A family-based and case-control association study of the dopamine D4 receptor gene and dopamine transporter gene in attention deficit hyperactivity disorder. Mol Psychiatry 2000; **5:** 523-30.

32. Mill J, Caspi A, Williams BS, Craig I, Taylor A, Polo-Tomas M, et al. Prediction of heterogeneity in intelligence and adult prognosis by genetic polymorphisms in the dopamine system among children with attention-deficit/hyperactivity disorder: Evidence from 2 birth cohorts. Arch Gen Psychiatry 2006; **63:** 462-9.

33. Curran S, Mill J, Sham P, Rijsdijk F, Marusic K, Taylor E, et al. QTL association analysis of the DRD4 exon 3 VNTR polymorphism in a population sample of children screened with a parent rating scale for ADHD symptoms. Am J Med Genet 2001; **105:** 387-93.

34. Payton A, Holmes J, Barrett JH, Hever T, Fitzpatrick H, Trumper AL, et al. Examining for association between candidate gene polymorphisms in the dopamine pathway and attention-deficit hyperactivity disorder: A family-based study. Am J Med Genet 2001; **105:** 464-70.

35. Holmes J, Payton A, Barrett J, Harrington R, McGuffin P, Owen M, et al. Association of DRD4 in children with ADHD and comorbid conduct problems. Am J Med Genet 2002; **114:** 150-3.

36. Paloyelis Y, Asherson P, Mehta MA, Faraone SV, Kuntsi J. DAT1 and COMT effects on delay discounting and trait impulsivity in male adolescents with attention deficit/hyperactivity disorder and healthy controls. Neuropsychopharmacology 2010; **35:** 2414-26.

37. Faraone SV, Biederman J, Weiffenbach B, Keith T, Chu MP, Weaver A, et al. Dopamine D4 gene 7-repeat allele and attention deficit hyperactivity disorder. Am J Psychiatry 1999; **156:** 768-70.

38. Comings DE, Gonzalez N, Wu S, Gade R, Muhleman D, Saucier G, et al. Studies of the 48 bp repeat polymorphism of the DRD4 gene in impulsive, compulsive, addictive behaviors: Tourette syndrome, ADHD, pathological gambling, and substance abuse. Am J Med Genet 1999; **88:** 358-68.

39. Barr CL, Wigg KG, Bloom S, Schachar R, Tannock R, Roberts W, et al. Further evidence from haplotype analysis for linkage of the dopamine D4 receptor gene and attention-deficit hyperactivity disorder. Am J Med Genet 2000; **96:** 262-7.

40. Lunetta KL, Faraone SV, Biederman J, Laird NM. Family-based tests of association and linkage that use unaffected sibs, covariates, and interactions. Am J Hum Genet 2000; **66:** 605-14.

41. McCracken JT, Smalley SL, McGough JJ, Crawford L, Del'Homme M, Cantor RM, et al. Evidence for linkage of a tandem duplication polymorphism upstream of the dopamine D4 receptor gene (DRD4) with attention deficit hyperactivity disorder (ADHD). Mol Psychiatry 2000; **5:** 531-6.

42. Todd RD, Neuman RJ, Lobos EA, Jong YJ, Reich W, Heath AC. Lack of association of dopamine D4 receptor gene polymorphisms with ADHD subtypes in a population sample of twins. Am J Med Genet 2001; **105:** 432-8.

43. Maher BS, Marazita ML, Ferrell RE, Vanyukov MM. Dopamine system genes and attention deficit hyperactivity disorder: A meta-analysis. Psychiatr Genet 2002; **12:** 207-15.

44. Smith KM, Daly M, Fischer M, Yiannoutsos CT, Bauer L, Barkley R, et al. Association of the dopamine beta hydroxylase gene with attention deficit hyperactivity disorder: Genetic analysis of the milwaukee longitudinal study. Am J Med Genet B Neuropsychiatr Genet 2003; **119B:** 77-85.

45. Kustanovich V, Ishii J, Crawford L, Yang M, McGough JJ, McCracken JT, et al. Transmission disequilibrium testing of dopamine-related candidate gene polymorphisms in ADHD: Confirmation of association of ADHD with DRD4 and DRD5. Mol Psychiatry 2004; **9:** 711-7.

46. Gornick MC, Addington A, Shaw P, Bobb AJ, Sharp W, Greenstein D, et al. Association of the dopamine receptor D4 (DRD4) gene 7-repeat allele with children with attention-deficit/hyperactivity disorder (ADHD): An update. Am J Med Genet B Neuropsychiatr Genet 2007; **144B:** 379-82.

47. Shaw P, Gornick M, Lerch J, Addington A, Seal J, Greenstein D, et al. Polymorphisms of the dopamine D4 receptor, clinical outcome, and cortical structure in attention-deficit/hyperactivity disorder. Arch Gen Psychiatry 2007; **64:** 921-31.

48. Lee SS, Humphreys KL. Interactive association of dopamine receptor (DRD4) genotype and ADHD on alcohol expectancies in children. Exp Clin Psychopharmacol 2014; **22:** 100-9.

49. Rowe DC, Stever C, Giedinghagen LN, Gard JM, Cleveland HH, Terris ST, et al. Dopamine DRD4 receptor polymorphism and attention deficit hyperactivity disorder. Mol Psychiatry 1998; **3:** 419-26.

50. Swanson JM, Sunohara GA, Kennedy JL, Regino R, Fineberg E, Wigal T, et al. Association of the dopamine receptor D4 (DRD4) gene with a refined phenotype of attention deficit hyperactivity disorder (ADHD): A family-based approach. Mol Psychiatry 1998; **3:** 38-41.

51. Grady DL, Chi HC, Ding YC, Smith M, Wang E, Schuck S, et al. High prevalence of rare dopamine receptor D4 alleles in children diagnosed with attention-deficit hyperactivity disorder. Mol Psychiatry 2003; **8:** 536-45.

52. Sunohara GA, Roberts W, Malone M, Schachar RJ, Tannock R, Basile VS, et al. Linkage of the dopamine D4 receptor gene and attention-deficit/hyperactivity disorder. J Am Acad Child Adolesc Psychiatry 2000; **39:** 1537-42.

53. Smalley SL, Bailey JN, Palmer CG, Cantwell DP, McGough JJ, Del'Homme MA, et al. Evidence that the dopamine D4 receptor is a susceptibility gene in attention deficit hyperactivity disorder. Mol Psychiatry 1998; **3:** 427-30.

54. Bidwell LC, Willcutt EG, McQueen MB, DeFries JC, Olson RK, Smith SD, et al. A family based association study of DRD4, DAT1, and 5HTT and continuous traits of attention-deficit hyperactivity disorder. Behav Genet 2011; **41:** 165-74.

55. Reiersen AM, Todorov AA. Association between DRD4 genotype and autistic symptoms in DSM-IV ADHD. J Can Acad Child Adolesc Psychiatry 2011; **20:** 15-21.

56. Frank Y, Pergolizzi RG, Perilla MJ. Dopamine D4 receptor gene and attention deficit hyperactivity disorder. Pediatr Neurol 2004; **31:** 345-8.

57. Castellanos FX, Lau E, Tayebi N, Lee P, Long RE, Giedd JN, et al. Lack of an association between a dopamine-4 receptor polymorphism and attention-deficit/hyperactivity disorder: Genetic and brain morphometric analyses. Mol Psychiatry 1998; **3:** 431-4.

58. Shahin O, Meguid NA, Raafat O, Dawood RM, Doss M, Bader El Din NG, et al. Polymorphism in variable number of tandem repeats of dopamine d4 gene is a genetic risk factor in attention deficit hyperactive egyptian children: Pilot study. Biomark Insights 2015; **10:** 33-8.

59. ElBaz Mohamed F, Kamal TM, Zahra SS, Khfagy MA, Youssef AM. Dopamine D4 receptor gene polymorphism in a sample of egyptian children with attention-deficit hyperactivity disorder (ADHD). J Child Neurol 2017; **32:** 188-93.

60. Tabatabaei SM, Amiri S, Faghfouri S, Noorazar SG, AbdollahiFakhim S, Fakhari A. DRD4 gene polymorphisms as a risk factor for children with attention deficit hyperactivity disorder in iranian population. Int Sch Res Notices 2017; **2017:** 2494537.

61. Eisenberg J, Zohar A, Mei-Tal G, Steinberg A, Tartakovsky E, Gritsenko I, et al. A haplotype relative risk study of the dopamine D4 receptor (DRD4) exon III repeat polymorphism and attention deficit hyperactivity disorder (ADHD). Am J Med Genet 2000; **96:** 258-61.

62. Kotler M, Manor I, Sever Y, Eisenberg J, Cohen H, Ebstein RP, et al. Failure to replicate an excess of the long dopamine D4 exon III repeat polymorphism in ADHD in a family-based study. Am J Med Genet 2000; **96:** 278-81.

63. Manor I, Tyano S, Eisenberg J, Bachner-Melman R, Kotler M, Ebstein RP. The short DRD4 repeats confer risk to attention deficit hyperactivity disorder in a family-based design and impair performance on a continuous performance test (TOVA). Mol Psychiatry 2002; **7:** 790-4.

64. Tahir E, Yazgan Y, Cirakoglu B, Ozbay F, Waldman I, Asherson PJ. Association and linkage of DRD4 and DRD5 with attention deficit hyperactivity disorder (ADHD) in a sample of turkish children. Mol Psychiatry 2000; **5:** 396-404.

65. Guney E, Iseri E, Ergun,S.G.,Percin,E.F., Ergun MA, Yalcin O, Sener S. The correlation of attention deficit hyperactivity disorder with DRD4 gene polymorphism in turkey. International Journal of Human Genetics 2013; **13:** 145-52.

66. Ercan ES, Suren S, Bacanli A, Yazici KU, Calli C, Ozyurt O, et al. Decreasing ADHD phenotypic heterogeneity: Searching for neurobiological underpinnings of the restrictive inattentive phenotype. Eur Child Adolesc Psychiatry 2016; **25:** 273-82.

67. Akay AP, Yazıcıoğlu CE, Güney SA, Erkuran HO, Kızıldağ S, Baykara B, et al. Allele frequencies of dopamine D4 receptor gene (DRD4) and catechol-O-methyltransferase (COMT) Val158Met polymorphism are associated with methylphenidate response in adolescents with attention deficit/hyperactivity disorder: A case control preliminary study. , Psychiatry and Clinical Psychopharmacology 2018; **28:** 177-84.

68. Roman T, Schmitz M, Polanczyk G, Eizirik M, Rohde LA, Hutz MH. Attention-deficit hyperactivity disorder: A study of association with both the dopamine transporter gene and the dopamine D4 receptor gene. Am J Med Genet 2001; **105:** 471-8.

69. Tovo-Rodrigues L, Rohde LA, Roman T, Schmitz M, Polanczyk G, Zeni C, et al. Is there a role for rare variants in DRD4 gene in the susceptibility for ADHD? searching for an effect of allelic heterogeneity. Mol Psychiatry 2012; **17:** 520-6.

70. Tovo-Rodrigues L, Rohde LA, Menezes AM, Polanczyk GV, Kieling C, Genro JP, et al. DRD4 rare variants in attention-deficit/hyperactivity disorder (ADHD): Further evidence from a birth cohort study. PLoS One 2013; **8:** e85164.

71. Carrasco X, Rothhammer P, Moraga M, Henriquez H, Aboitiz F, Rothhammer F. Presence of DRD4/7R and DAT1/10R allele in chilean family members with attention deficit hyperactivity disorder. Rev Med Chil 2004; **132:** 1047-52.

72. Carrasco X, Rothhammer P, Moraga M, Henriquez H, Chakraborty R, Aboitiz F, et al. Genotypic interaction between DRD4 and DAT1 loci is a high risk factor for attention-deficit/hyperactivity disorder in chilean families. Am J Med Genet B Neuropsychiatr Genet 2006; **141B:** 51-4.

73. Henriquez-Henriquez M, Villarroel L, Henriquez H, Zamorano F, Rothhammer F, Aboitiz F. Intratask variability as a correlate for DRD4 and SLC6A3 variants: A pilot study in ADHD. J Atten Disord 2015; **19:** 987-96.

74. Arcos-Burgos M, Castellanos FX, Konecki D, Lopera F, Pineda D, Palacio JD, et al. Pedigree disequilibrium test (PDT) replicates association and linkage between DRD4 and ADHD in multigenerational and extended pedigrees from a genetic isolate. Mol Psychiatry 2004; **9:** 252-9.

75. Fonseca DJ, Mateus HE, Galvez JM, Forero DA, Talero-Gutierrez C, Velez-van-Meerbeke A. Lack of association of polymorphisms in six candidate genes in colombian adhd patients. Ann Neurosci 2015; **22:** 217-21.

76. Gabriela ML, John DG, Magdalena BV, Ariadna GS, Francisco de LP, Liz SM, et al. Genetic interaction analysis for DRD4 and DAT1 genes in a group of mexican ADHD patients. Neurosci Lett 2009; **451:** 257-60.
