# Supplementary material for: DRD4 48 bp multiallelic variants as age-population-specific biomarkers in attention-deficit/hyperactivity disorder
Source: Transl Psychiatry. 2020 Feb 19;10:70. doi: 10.1038/s41398-020-0755-4 (PMC7031506; doi:10.1038/s41398-020-0755-4)
Supplement: Supplementary file 14 — Supplementary material Legends [file 41398_2020_755_MOESM14_ESM.docx]

**Supplementary material**

**Legends’s Figures**

**Fig. S1**

PRISMA Flow chart depicting the selection procedure for review and meta-analyses.

**Fig. S2**

Random forest plot for Odds Ratio from meta-analyses of CC (case-control) and TDT (Transmission Disequilibrium Test) studies of allele 2R of 48bp VNTR in *Dopamine receptor D4* (*DRD4)* gene in different populations (ADHD childhood).

Captions: Chi2=χ2 test of goodness of fit; Tau2=estimate of the between-study variance in a random-effects meta-analysis.

**Fig. S3**

Random forest plot for Odds Ratio from meta-analyses of CC (case-control) and TDT (Transmission Disequilibrium Test) studies of allele 4R of 48bp VNTR in *Dopamine receptor D4* (*DRD4)* gene in different populations (ADHD childhood).

Captions: Chi2=χ2 test of goodness of fit; Tau2=estimate of the between-study variance in a random-effects meta-analysis.

**Fig. 4S**

Random forest plot for Odds Ratio from meta-analyses of CC (case-control) and TDT (Transmission Disequilibrium Test) studies of allele 7R of 48bp VNTR in *Dopamine receptor D4* (*DRD4)* gene in different populations (ADHD childhood).

Captions: Chi2=χ2 test of goodness of fit; Tau2=estimate of the between-study variance in a random-effects meta-analysis.

**Fig. 5S**

Random forest plot for Odds Ratio from meta-analyses of CC (case-control) and TDT (Transmission Disequilibrium Test) studies of long allele of 48bp VNTR in *Dopamine receptor D4* (*DRD4)* gene in different populations (ADHD childhood).

Captions: Chi2=χ2 test of goodness of fit; Tau2=estimate of the between-study variance in a random-effects meta-analysis.

**Fig. 6S**

Random forest plot for Odds Ratio from *in vitro* studies related to test the associations with the functionality of 48bp VNTR in *Dopamine receptor D4* (*DRD4)* gene: allele 2R versus allele 4R.

Captions: Chi2=χ2 test of goodness of fit; Tau2=estimate of the between-study variance in a random-effects meta-analysis.

**Fig. 7S**

Random forest plot for Odds Ratio from *in vitro* studies related to test the associations with the functionality of 48bp VNTR in *Dopamine receptor D4* (*DRD4)* gene: allele 2R versus allele 7R.

Captions: Chi2=χ2 test of goodness of fit; Tau2=estimate of the between-study variance in a random-effects meta-analysis.

**Fig. 8S**

Random forest plot for Odds Ratio from *in vitro* studies related to test the associations with the functionality of 48bp VNTR in *Dopamine receptor D4* (*DRD4)* gene: allele 4R versus allele 7R.

Captions: Chi2=χ2 test of goodness of fit; Tau2=estimate of the between-study variance in a random-effects meta-analysis.

**Fig. 9S**

*Dopamine receptor D4* (*DRD4)* gene Linkage Disequilibrium blocks for different populations (African, American, East Asian, European and South Asian).

**Table S1.**

Results of the Egger’s test for publication bias.

**Table S2.**

Results of the Newcastle-Ottawa Scale for 48bp VNTR in *Dopamine receptor D4* (*DRD4)* gene.

**Supplementary References Table 1**

References for Table 1.

**Supplementary References Table 4**

References for Table 4.
